# Dissecting the role of *comS*-independent *srf* expression on multicellular differentiation and competence development in *Bacillus subtilis*

**DOI:** 10.3389/fmicb.2026.1753310

**Published:** 2026-02-04

**Authors:** Sarah Miercke, Jan-Philipp Knepper, Klaus Dreisewerd, Thorsten Mascher

**Affiliations:** 1 TUD Dresden University of Technology, Chair of General Microbiology, Dresden, Germany; 2 Institute of Hygiene, University of Münster, Münster, Germany

**Keywords:** *Bacillus subtilis*, biofilm, competence, gene expression, MALDI-MS imaging, multicellular differentiation, stop mutations, surfactin

## Abstract

In its natural soil habitat, *B. subtilis* regularly encounters fluctuating conditions that require adaptive survival strategies, including the production and secretion of antimicrobial compounds. One such compound, surfactin, play a central role in multicellular differentiation processes such as biofilm formation, swarming, and competence development. Competence and surfactin biosynthesis are transcriptionally co-regulated via the quorum sensing-mediated activation of the *srfAABCD* operon, which contains *comS* in a distinct open reading frame overlapping with *srfAB*. This study aimed at uncoupling competence from surfactin production by introducing targeted stop mutations in *comS* to selectively disrupt competence without affecting surfactin synthesis. For this, we introduced single nucleotide polymorphisms (SNPs) that preserved the *srfAB* codons, while simultaneously introducing a premature stop codon in *comS*. The effects on competence development were assessed using luciferase-based reporter assays monitoring the ComS-dependent expression of *comK* and *comGA* expression. Surfactin production was analyzed by mass spectrometry imaging and phenotypic assays examining the impact on multicellular behavior. Our findings demonstrate that the generated point mutations severely reduce competence gene expression, measured via P*_comK_* and P*_comGA_* activity, to levels comparable with a full *comS* deletion, while leaving multicellular behaviors such as biofilm and pellicle formation, as well as swarming and sliding motility, unaffected. Thus, ComS is specifically essential for competence development but dispensable for other surfactin-mediated multicellular processes and not involved in structuring biofilms. Taken together, our results demonstrate that it is possible to genetically decouple competence from other developmental pathways in *B. subtilis*.

## 1 Introduction

*Bacillus subtilis*, particularly the undomesticated strain NCIB3610 (Branda et al., 2001) and its genetic accessible equivalent DK1042 (Konkol et al., 2013), serve as model organisms for investigating cellular differentiation and underlying regulatory networks, including competence development, resulting in the ability to take up extracellular DNA from its environment, and multicellular behavior (Vlamakis et al., 2013). The soil, as its major habitat, is characterized by dynamic and fluctuating environmental conditions that require adaptation and survival strategies through bacterial warfare to prevail against competitors in their own niche. The production of antibiotics represents a key strategy for defending against external threats and is enhanced by the formation of biofilms, which facilitate the synchronization of signaling pathways, and the orchestration of stress response mechanisms associated with cellular differentiation (Camilli and Bassler, 2006). Within biofilms, the division of labor among specialized subpopulations enhances community adaptability, while the surrounding extracellular matrix provides additional protection (Vlamakis et al., 2013; Vlamakis et al., 2008).

One of the subpopulations in *B. subtilis* biofilms are the surfactin-producing cells. Surfactin is a multifunctional antimicrobial lipopeptide with both ecological and industrial significance, since it is used in e. g. food processing and preservation (Wahab and Al-Sahlany, 2025). It has also been reported to exhibit antiviral and antitumoral properties, making it an attractive candidate for biomedical applications such as drug delivery systems (Wahab and Al-Sahlany, 2025; Yadav and Eswari, 2022). Ecologically, surfactin reduces surface tension, facilitates rhizosphere colonization (Aleti et al., 2016), and contributes to the biocontrol of plant pathogens (Bais et al., 2004). As a potent biosurfactant, surfactin is essential for biofilm formation and drives the emergence of swarming, a flagella-dependent movement, as well as sliding, a flagella-independent spreading (Kearns and Losick, 2003). Moreover, it is implicated in the regulation of diverse (multicellular) differentiation processes, including cannibalism, sporulation and competence (López et al., 2009a; López et al., 2009c).

**Figure 1:**
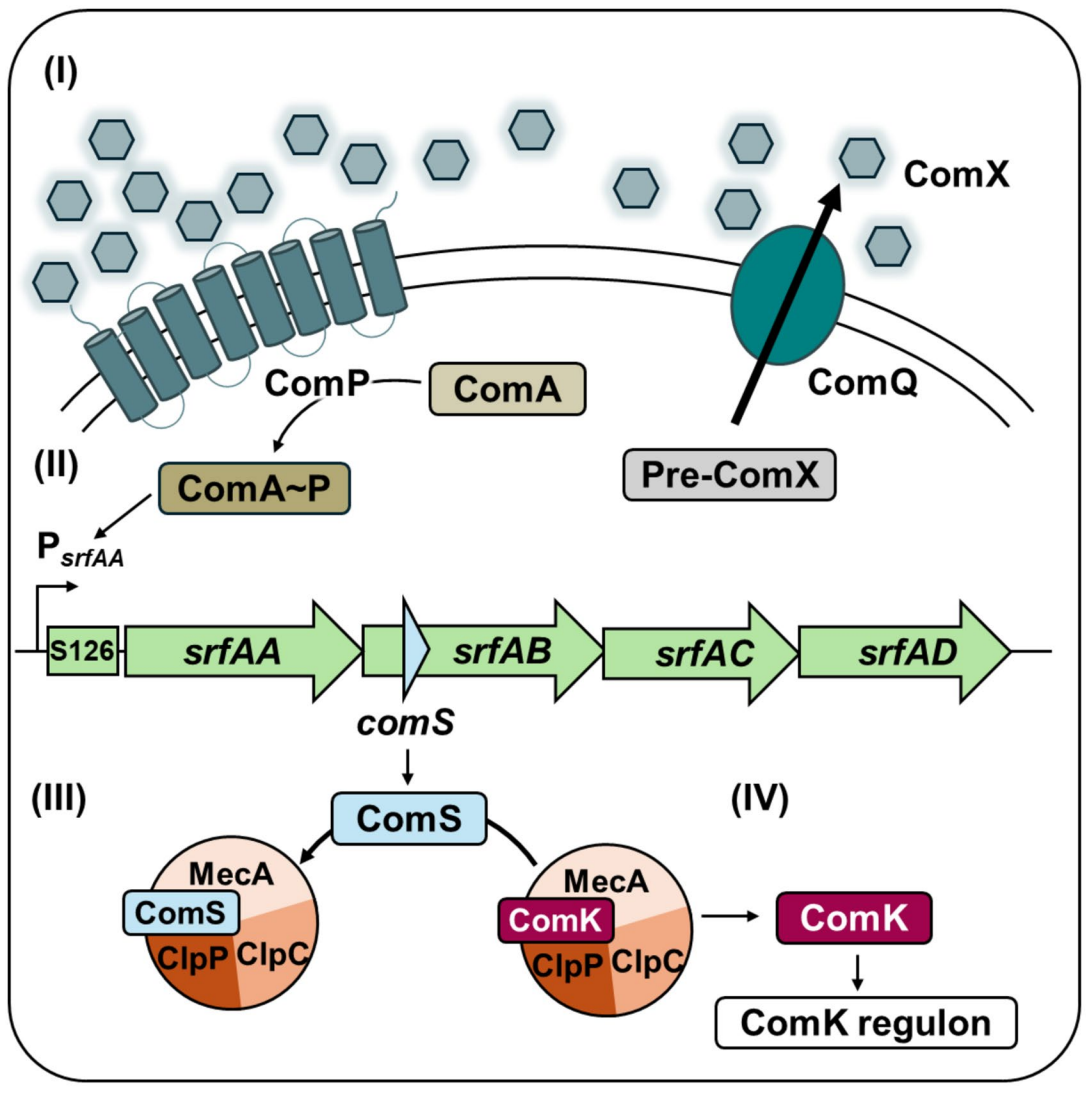
Competence development in *B. subtilis*. (I) Under nutrient limitation and high cell density, the quorum sensing peptide ComX is sensed by the two-component system ComP-ComA, leading to ComA phosphorylation (ComA~P). (II) ComA~P activates transcription of the *srfAABCD* operon, which also encodes *comS*. (III) The small competence protein ComS protects ComK, the master regulator of competence, from degradation by the MecA/ClpCP protease complex. (IV) Once ComK reaches a threshold, it activates the ComK regulon, expressing proteins required for DNA uptake, enabling transient genetic competence in a subpopulation of cells.

The regulatory link between competence and surfactin production is embedded in their genetic organization. Surfactin biosynthesis is based on expression of the approximately 27 kb large *srfAABCD* (*srf*) operon (Cosmina et al., 1993), which also encodes the small competence protein ComS within *srfAB* in a distinct open reading frame (Figure 1). Expression of the operon is driven by the σ^D^-dependent P*_srfAA_* promoter and activated through quorum sensing-dependent phosphorylation of ComA via the ComQXPA system (Nakano et al., 1991). ComX functions as a competence pheromone, which is processed and exported by ComQ to then be sensed by the two-component system ComPA consisting of the sensor histidine kinase ComP, and the transcriptional regulator ComA (Figure 1, I). Upon accumulation of ComX, ComP phosphorylates ComA that subsequently binds to P*_srfAA_* region leading to initiation of transcription of both *srfAABCD* and *comS* (Figure 1, II) (Comella and Grossman, 2005). Subsequently, ComS prevents proteolytic degradation of ComK, the master regulator of competence, by competitively binding the MecA/ClpCP proteolytic complex (Figure 1, III), ultimately triggering expression of ComK and hence competence development (Figure 1, IV) (D'Souza et al., 1994).

**Figure 2:**
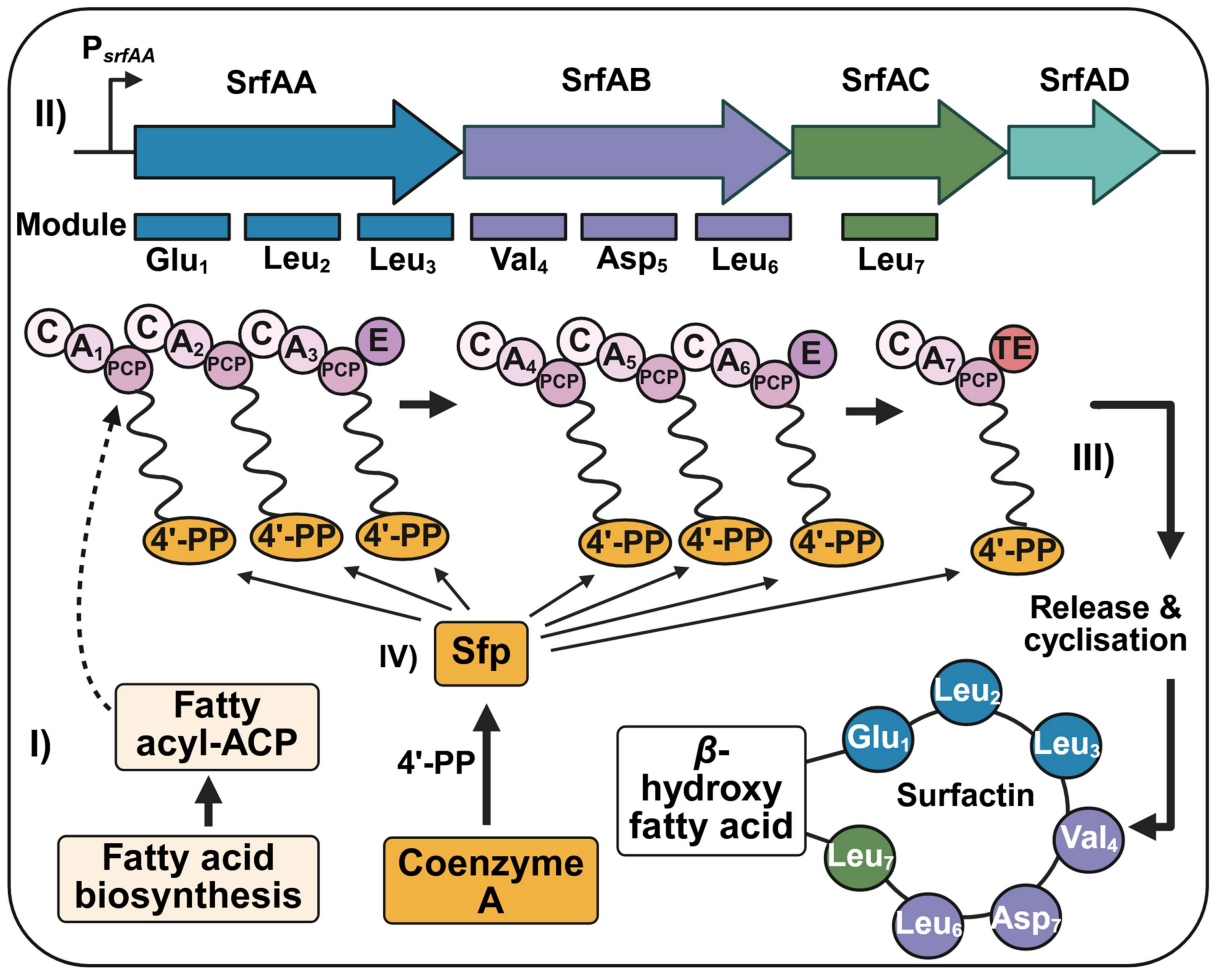
Non-ribosomal surfactin biosynthesis in *B. subtilis*. (I) Surfactin biosynthesis is initiated by transferring a *β*-hydroxy fatty acid, derived from fatty acid biosynthesis as fatty acyl-acyl carrier protein (ACP) intermediates, onto the first peptidyl carrier protein (PCP) domain of the non-ribosomal peptide synthetase (NRPS) complex. (II) The NRPS complex, encoded by *srfAA, srfAB*, and *srfAC*, incorporates the seven amino acids of surfactin (Glu-Leu-Leu-Val-Asp-Leu-Leu) through a modular arrangement of condensation (C), adenylation (A), and PCP domains. Epimerization (E) domains at the C-terminus of SrfAA and SrfAB introduce D-amino acids, while the covalently linked type I thioesterase (TE) domain at the C-terminus of SrfAC catalysis the peptide chain release and cyclisation. The macrolactone formation occurs between the *β*-hydroxy fatty acid and Leu_7_. SrfAD acts as a proofreading enzyme, recycling misacylated PCP domains. (IV) Further, *sfp* encodes for a phosphopantetheinyl transferase and is required for post-translational activation of surfactin. Sfp phosphopantetheinylates a serine residue in each PCP domain. The 4′-phosphopantetheine (4’-PP) is derived from coenzyme A.

Beyond its role in competence regulation, the *srf* operon also encodes the machinery for surfactin biosynthesis, which is initiated by transferring a *β*-hydroxy fatty acid, derived from fatty acid biosynthesis as fatty acyl-acyl carrier protein (ACP) intermediates, onto the first peptidyl carrier protein (PCP) domain of the non-ribosomal peptide synthetase (NRPS) complex (Figure 2, I; Nakano et al., 1988; Kraas et al., 2010). The NRPS complex consists of three large subunits (SrfAA, SrfAB, and SrfAC) and the type II thioesterase SrfAD that assemble the cyclic heptalipopeptide (L-Glu_L-Leu_D-Leu_L-Val_L-Asp_D-Leu_L-Leu) (Nakano et al., 1988; Vater et al., 1997). Each NRPS module is responsible for incorporating one amino acid into the peptide chain and consists of several key domains, including the adenylation (A) domain, condensation (C) domain, and peptidyl carrier protein (PCP) domain (Figure 2, II; Nakano et al., 1988; Kraas et al., 2010). Further, two epimerization (E) domains, located at the C-terminus of SrfAA and SrfAB as well as a covalently linked type I thioesterase (TE) domain at the C-terminus of SrfAC are part of the NRPS complex (Figure 2, II; Nakano et al., 1988; Mootz and Marahiel, 1997; Marahiel et al., 1997). The type I thioesterase catalyzes the macrolactone formation between Leu_7_ and the *β*-hydroxy fatty acid and subsequently releases the peptide (Figure 2, III; Tanovic et al., 2008; Koglin et al., 2008). SrfAD, an external type II thioesterase, acts as a proofreading enzyme to recycle, e. g. misacetylated PCP domains, which block the reactive thiol group of the 4′-phosphopantetheine (4’-PP) cofactor attached to the PCP domain (Kraas et al., 2010; Koglin et al., 2008; Schwarzer et al., 2002). Further, surfactin production requires post-translational activation by the phosphopantetheinyl transferase Sfp, which phosphopantetheinylates a serine residue in each PCP domain. The transferred 4’-PP is derived from coenzyme A (Figure 2, IV; Cosmina et al., 1993; Quadri et al., 1998). Active surfactin contributes to lowering surface tension and promoting swarming motility, while simultaneously displaying antimicrobial effects (Buchoux et al., 2008; Kinsinger et al., 2003). It disrupts membranes and forms pores, causing potassium leakage, a known trigger for activating sporulation-associated histidine kinases (Kinsinger et al., 2003). As a consequence, surfactin indirectly supports both sporulation initiation and competence (Nakano et al., 1991).

While the genetic coupling of *comS* and *srfAABCD* is well established, their individual contributions to multicellular differentiation remain poorly understood. In this study, we aimed at disentangling this regulatory link by introducing target-specific stop mutations in *comS*, enabling *srfAABCD* expression in the absence of ComS. The functionality of these mutations was evaluated by monitoring *comK* and *comGA* expression, which serve as markers of competence development. Additionally, microbial matrix-assisted laser desorption/ionization mass spectrometry imaging (MALDI-MSI) was used to confirm that surfactin production was unaffected (Friebel et al., 2025; Bourceau et al., 2023). This approach allowed dissecting the specific roles of competence and surfactin production in shaping multicellular differentiation.

## 2 Materials and methods

### 2.1 Bacterial strains and general culture conditions

All strains (Supplementary Table S1) were, if not indicated otherwise, routinely cultivated at 37 °C with agitation (180 rpm) in lysogeny broth (LB) media (10 g/L tryptone, 5 g/L yeast extract, 10 g/L NaCl). LB agar plates were prepared by the addition of 1.5% (w/v) agar-agar. Selection of *B. subtilis* strains harboring a resistance marker were carried out by using 5 μg/mL chloramphenicol, 10 μg/mL kanamycin, 1 μg/mL erythromycin along with 25 μg/mL lincomycin for MLS, or a combination of these antibiotics, respectively. For cultivation of *E. coli* strains carrying a plasmid 100 μg/mL ampicillin or 50 μg/mL kanamycin were used. Long time storage at −70 °C was achieved by supplementing the overnight culture with 50% (v/v) glycerol at a ratio of 1:1.

### 2.2 DNA manipulation

All restriction endonucleases, T4 DNA ligase, Q5 DNA polymerase, and OneTaq® DNA polymerase originated from New England Biolabs (Ipswich, MA, United States) and cloning procedures followed the respective protocols. DNA fragments were purified using Hi Yield® PCR Clean-up/ Gel Extraction kit (Süd-Laborbedarf GmbH, Gauting, Germany) or NucleoSpin® Gel and PCR Clean-up kit (Macherey-Nagel, Düren, Germany) and plasmids were isolated applying Hi Yield® Plasmid mini kit (Süd-Laborbedarf GmbH, Gauting, Germany) according to the corresponding manufacturer’s recommendations. Genomic DNA of *B. subtilis* was isolated either with the NucleoSpin® Microbial DNA kit (Macherey-Nagel, Düren, Germany) used as PCR template or by SC lysis for transformation (Harwood, 1990). Transformation of *E. coli* and *B. subtilis* was carried out as described previously (Harwood, 1990; Sambrook and Russell, 2001)^.^ Successful genetic or genomic modification was verified by colony PCR using OneTaq® DNA polymerase and sequencing. Plasmids and oligonucleotides used were listed in Table S2 and Table S3, respectively.

### 2.3 Construction of plasmids

Targeted introduction of single nucleotide polymorphisms (SNPs), which resulted in stop codons within *comS*, was carried out using the CRISPR-Cas9 vector pJOE8999 (Altenbuchner, 2016), which contains the *cas* gene under control of a mannose-inducible promoter (P*_manP_*), a customizable guide RNA (gRNA) module driven by the semi-synthetic P*_vanP_* promoter, a kanamycin resistance marker, and dual origins of replication (*oriR*): one from plasmid pUC19 for replication in *E. coli* and pET194^ts^, a temperature sensitive *oriR* for replication in *B. subtilis* (Supplementary Figure S1a).

To generate the gRNA expression construct (pJOE8999_gRNA), 5′-phosphorylated oligonucleotides TM7980 and TM7981 purchased from Sigma-Aldrich (St. Louis, Missouri (MO), USA) were annealed by heating to 98 °C for 3 min and cooling to 25 °C at 0.2 °C/s in 1X r2.1 buffer (NEB, Ipswich, MA, United States). The resulting double-stranded DNA was ligated into BsaI-digested pJOE8999 using golden gate assembly (Engler et al., 2008; Supplementary Figure S1b). The reaction (10 μL) contained 50 ng vector, 1 μL BsaI, 1 μL T4 DNA ligase, 1 μL ligase buffer, 0.2 μL bovine serum albumin (BSA), and 5 μL of annealed oligonucleotides. Thirty cycles of digestion (37 °C, 5 min) and ligation (16 °C, 10 min) were followed by final digestion (37 °C, 1 h) and enzyme inactivation (80 °C, 10 min). The entire reaction was applied for transformation of *E. coli* DH10*β* and correct gRNA insertion was confirmed. The verified pJOE8999_gRNA plasmid was digested with SalI and Xbal, and the vector backbone was gel-purified from 1% (w/v) agarose 1X TAE gel (1 mM EDTA disodium salt, 40 mM TRIS, 20 mM acetic acid). Following, homology-directed repair (HDR) templates were assembled from PCR-amplified upstream and downstream regions flanking the *comS* target site, as well as a central fragment containing the desired SNP and a modified protospacer adjacent motif (PAM) sequence to prevent re-cleavage by Cas9 (Supplementary Figure S1c).

All fragments were amplified with oligonucleotides containing BsaI recognition sites. The use of BsaI as type IIS restriction enzyme that cleaves outside of its recognition site, enabled control over the resulting DNA overhangs and allowed markerless assembly of the HDR fragments while simultaneously introducing the desired point mutations including stop-gain SNP and PAM sequence alteration (Table 1; Supplementary Figure S1d).

**Table 1:** Oligonucleotides applied for the generation of pJOE8999 derivate plasmids.

Plasmid	Oligonucleotides	DNA fragment*
pJOE8999_gRNA_*comS* C11A	TM7965 & TM7967	C11A upstream
	TM7968 & TM7982	C11A PAM exchange
	TM7983 & TM7966	C11A downstream
pJOE8999_gRNA_*comS* C74A	TM7965 & TM7969	C74A upstream
	TM7970 & TM7982	C74A PAM exchange
	TM7983 & TM7966	C74A downstream
pJOE8999_gRNA_*comS* C74G	TM7965 & TM7971	C74G upstream
	TM7972 & TM7982	C74G PAM exchange
	TM7983 & TM7966	C74G downstream
pJOE8999_gRNA_*comS* T83A	TM7965 & TM7973	T83A upstream
	TM7974 & TM7982	T83A PAM exchange
	TM7983 & TM7966	T83A downstream
pJOE8999_gRNA_*comS* G128A	TM7965 & TM7975	G128A upstream
	TM7976 & TM7982	G128A PAM exchange
	TM7983 & TM7966	G128A downstream

*PAM = Protospacer adjacent motif.

Subsequently, golden gate assembly was performed to insert the HDR fragments into the vector using a 1:10 molar ratio of vector to each fragment (upstream, PAM exchange, and downstream part). Following, the constructed plasmids (pJOE8999_gRNA_*comS* C11A, C74A, C74G, T83A, and G128A) were used to transform *B. subtilis*. Transformants were selected on LB agar plates supplemented with kanamycin and 0.2% (w/v) mannose to induce Cas9 expression. Plates were incubated at 30 °C to maintain replication by the temperature-sensitive pE194^ts^ *oriR*. Colonies harboring the plasmid were re-streaked on LB agar plates without antibiotics and incubated at 50 °C overnight to promote plasmid backbone loss. Single colonies were patched onto LB agar plates with and without kanamycin, and screened for kanamycin sensitivity. The presence of the desired point mutations was verified by sequencing.

Luciferase-based reporter and implementation strains were constructed following the protocols described by Radeck et al. (2013) and Popp et al. (2017). Transcriptional *luxABCDE* (*lux*) fusions were generated using the vector pBS3C*lux*, into which the desired promoter fragments were inserted. The P*_comGA_*, P*_comK_*, and P*_srfAA_* DNA fragments were amplified by using the oligonucleotides TM8067 & TM8068, TM7811 & TM7812, and TM7512 & TM7514, respectively.

### 2.4 Analysis of multicellular and motility behavior of *Bacillus subtilis*

The minimal medium MSggN (5 mM sodium phosphate buffer pH 7.0; 100 mM MOPS; 2 mM MgCl_2_; 700 μM CaCl_2_; 50 μM MnCl_2_; 700 μM FeCl_3_; 1 μM ZnCl_2_; 2 μM thiamine hydrochloride; 0.5% (v/v) glycerol; 0.5% (w/v) K-glutamate; 100 μM KCl) (Fall et al., 2006), a modified MSgg medium (Branda et al., 2001), was used to analyze sliding motility. For solidification, 0.35% (w/v) agarose were added to the MSggN broth. The original MSgg medium (5 mM potassium phosphate buffer pH 7.0; 100 mM MOPS; 2 mM MgCl_2_; 700 μM CaCl_2_; 50 μM MnCl_2_; 700 μM FeCl_3_; 1 μM ZnCl_2_; 2 μM thiamine hydrochloride; 0.5% (v/v) glycerol; 0.5% (w/v) K-glutamate; 50 μg/mL tryptophan, 50 μg/mL phenylalanine) was selected to form biofilms and pellicles. For pellicle generation 6 mL MSgg broth were transferred into each well of a 6-well plate (Greiner Bio-One, Frickenhausen, Germany). Biofilms were grown on MSgg plates solidified with 1.5% (w/v) agar-agar. Swarming behavior was investigated using LB soft agar plates with a final concentration of 0.7% (w/v) agar-agar. All agar plates were allowed to dry for at least 45 min before usage. For inoculation of the different media *B. subtilis* strains were standardized cultivated overnight without the addition of antibiotics. Pellicle formation was carried out by inoculation of MSgg broth to final optical density at 600 nm wavelength (OD_6oo_) of 0.1 and incubation at 25 °C for 5 days.

All solid differentiation agar plates were inoculated at the center of the plate with 5 μL of cell suspension, which was previously diluted to OD_600_ 0.1 with LB broth. While LB soft-agar plates (swarming assay), and MSggN agar plates (sliding assay) were incubated at 30 °C for 24 h, the MSgg agar plates (biofilm assay) were grown at 25 °C for 7 days. Digital documentation of the resulting morphologies was performed with either the PCAM camera (TU Dresden) or the binocular microscope WILD M3Z (Leica Microsystems GmbH, Wetzlar, Germany) with a ProgRes SpeedXT ^core^®5 camera (Jenoptik, Jena, Germany).

### 2.5 Bacterial luciferase assay

The luciferase activity of the strains carrying promoter *luxABCDE* fusions (P*_comK_*-*lux*; P*_srfAA_*-*lux*; P*_comGA_*-*lux*) was analyzed using the Synergy Neo 2 multi-mode microplate reader connected to the BioSpa 8 automated incubator from Agilent Technologies (Santa Clara, CA, United States), and controlled by the Gen5™ software. The assays were undertaken in 96-well plates (black walls, clear bottom, Greiner Bio-One, Frickenhausen, Germany) and cell growth was monitored at OD_600_. Relative luminescence units (RLU) were measured and normalized to the optical density, whereby each RLU data point was divided by the corresponding OD_600_ (RLU/OD_600_). Strains were routinely cultivated overnight and used for inoculation of 5 mL minimal medium MNGE (2% (w/v) glucose; 0.2% (w/v) glutamate; 11 μg/mL Fe(III)-ammonium citrate; 3 mM MgSO_4_; 92% (w/v) 1X MN) to an OD_600_ of 0.1. Subsequently, the culture was grown at 37 °C with agitation (180 rpm) till OD_600_ 0.2–0.4 and diluted to OD_600_ 0.05. Following, 100 μL of the cell suspension were transferred into the 96-well plate and measurements were performed at 37 °C, whereby the plated was linear shaken for 10 s before each measurement. The OD_600_ and luminescence were monitored every 9 min for at least 18 h. Data analysis and preparation of graphs was carried out by using GraphPad Prism 5.0 software (GraphPad, San Diego, CA, United States).

### 2.6 Matrix-assisted laser desorption/ionization mass spectrometry imaging

Biofilms were grown on mixed cellulose ester membranes with 0.22 μm average pore size and a thickness of 150 μm following the procedure described earlier (Friebel et al., 2025). The membranes were used to ensure easy detachment of the biofilms from the MSgg agar and to simplify subsequent inactivation as described previously (Friebel et al., 2025; Brockmann et al., 2019). For fixation and safe inactivation, bacterial biofilms were cut out and wetted with a solution of 10% (v/v) formaldehyde (Carl Roth GmbH & Co. KG, Karlsruhe, Germany) in water. After 30 min, fixation was complete and the biofilms were washed two times with Milli-Q water (Merck KGaA, Darmstadt, Germany) and then glued together with the membrane on a microscope slide using super glue (UHU GmbH & Co KG, Bühl, Germany). A VS200 microscope slide scanner with a 4X objective (Evident GmbH, Hamburg, Germany) and ORCA-Fusion C14440 20UP camera (Hamamatsu photonics, Hamamatsu City, Japan) was used for reflected light microscopy of biofilms. For microscope image visualization, OlyVia (version 3.4.1, Evident, Hamburg, Germany) was used. A solution of 7 mg/mL 2,5-dihydroxyacetophenone (Merck KgaA, Darmstadt, Germany) dissolved in 75% (v/v) acetonitrile (Carl Roth GmbH & Co. KG, Karlsruhe, Germany), 10% (v/v) methanol (Carl Roth GmbH & Co. KG, Karlsruhe, Germany), 10% (v/v) trifluoroacetic acid (Carl Roth GmbH & Co. KG, Karlsruhe, Germany) and 5% (v/v) Milli-Q water was sprayed on the biofilm as MALDI matrix using a SunCollect pneumatic spray roboter (SunChrom, Friedrichsdorf, Germany). The matrix was applied within 22 spraying cycles at a nitrogen back pressure of 2.5 bar in a meandering pattern. The flow rate followed a gradient and started at 15 μL/min in the first cycle and increased to 20 μL/min and 30 μL/min for the second and third spraying cycle, respectively. From the fourth spraying cycle onwards, the flow rate was 50 μL/min until the end. The spray nozzle moved with a velocity of 700 mm/min and a line distance of 2 mm. The distance between the spray nozzle and sample surface was set to 44 mm.

All MALDI-MSI measurements were conducted with a timsTOF fleX MALDI-2 QTOF mass spectrometer (Bruker Daltonics GmbH & Co. KG, Bremen, Germany), equipped with a Smartbeam3D™ laser emitting at 355 nm providing a quadratic focal spot size of 5 × 5 μm diameter. For all imaging experiments, the resulting field size was 50 μm using beam scan function and the “M5 small” setting with 200 laser shots per pixel with a frequency of 1 kHz and a nitrogen partial pressure in the MALDI source of 2.5 mbar. With the analytical focus on the detection of surfactin along with the peptide toxins EPE, SKF, and SDP, all herein reported MSI data were acquired in the “high sensitivity detection” mode within a *m*/*z* range of 1,000–4,500 in positive ion mode and with the MALDI-2-postionisation disabled. MSI data was processed using SCiLS Lab MVS (vs. 2026a Pro, Bruker Daltonics GmbH & Co. KG, Bremen, Germany) and ion images visualized as false color images using the same software. MSI experiments were performed in biological and technical duplicates.

## 3 Results

### 3.1 ComS truncation uncouples competence from Surfactin production

**Figure 3:**
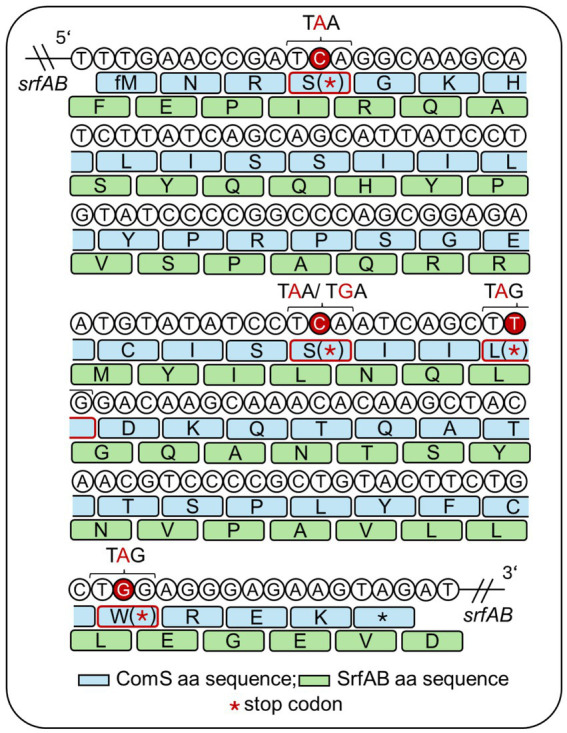
Schematic overview of the point mutations introduced in the *srf* operon. *ComS* is encoded within the *srf* operon, which expression is driven by the *srfAA* promoter. The position of the introduced point mutations within the ComS (blue) and SrfAB (green) amino acid sequence are highlighted. The target specific stop-gained single nucleotide polymorphisms were introduced to selectively disrupt competence development while maintaining surfactin biosynthesis, enabling a functional dissection of *comS*-mediated signaling. While the altered nucleotides are highlighted in red, the amino acids of *ComS* changed to a stop codon (red asterisk) are marked in red boxes. Five different point mutations were generated by CRISPR-Cas9 based approach: *ComS* C11A, C74A, C74G, T83A, and G128A.

In *B. subtilis*, competence development and surfactin production are genetically coupled though co-transcription of *comS* and *srfAB*. However, their gene products exhibit distinct physiological roles. While ComS stabilizes ComK by preventing its proteolytic degradation, initiating the competence pathway (Figure 1), *srfAB* encodes components for the non-ribosomal peptide synthetase complex responsible for surfactin biosynthesis (Figure 2). To investigate the role of surfactin independent of *comS* expression, we introduced targeted single nucleotide polymorphisms (SNPs) that lead to premature stop codons within the *comS* coding sequence (Figure 3).

These point mutations were strategically selected to truncate *comS* translation while preserving the amino acid sequence of the overlapping *srfAB*. Five distinct mutations within *comS* - 11C > A (Ser4*), 74C > A (Ser25*), 74C > G (Ser25*), 83 T > A (Leu28*), and 128G > A (Trp43*) - hereafter referred to as *comS* C11A, C74A, C74G, T83A, and G128A, were introduced into the *comS* coding sequence by CRISPR-Cas9-based genome editing, employing the vector pJOE8999 (Altenbuchner, 2016; Figure 3). This system allowed precise incorporation of the desired point mutations into the homology repair templates, which mediated repair of the Cas9-induced double-strand break at the target site within *srfAB* (Supplementary Figure S1). All mutations occur within codons that encode either isoleucine or leucine in the overlapping *srfAB* gene, allowing synonymous substitutions that preserve SrfAB protein function. Codon usage at the mutated sites in *srfAB* was evaluated based on Nakamura et al. (2000) to ensure translational efficiency was not compromised (Table S4). Although codon usage frequencies were either slightly reduced (C11A, C74G, T83A, and G128A) or modestly increased (C74A) compared to the wild type, all resulting codons retained a usage frequency of at least approximately 5 per thousand, suggesting they remain within the range of commonly used codons in *B. subtilis* (Nakamura et al., 2000). A summary of codon usage before and after mutagenesis is provided in Table S4. The mutations resulted in truncated ComS peptides of varying lengths: C11A resulted in the shortest version with 2 amino acids, whereas G128A led to almost complete translation of ComS lacking only the final three residues (Figure 3).

### 3.2 Impact of *coms* and *srf* mutations on multicellular behavior

**Figure 4:**
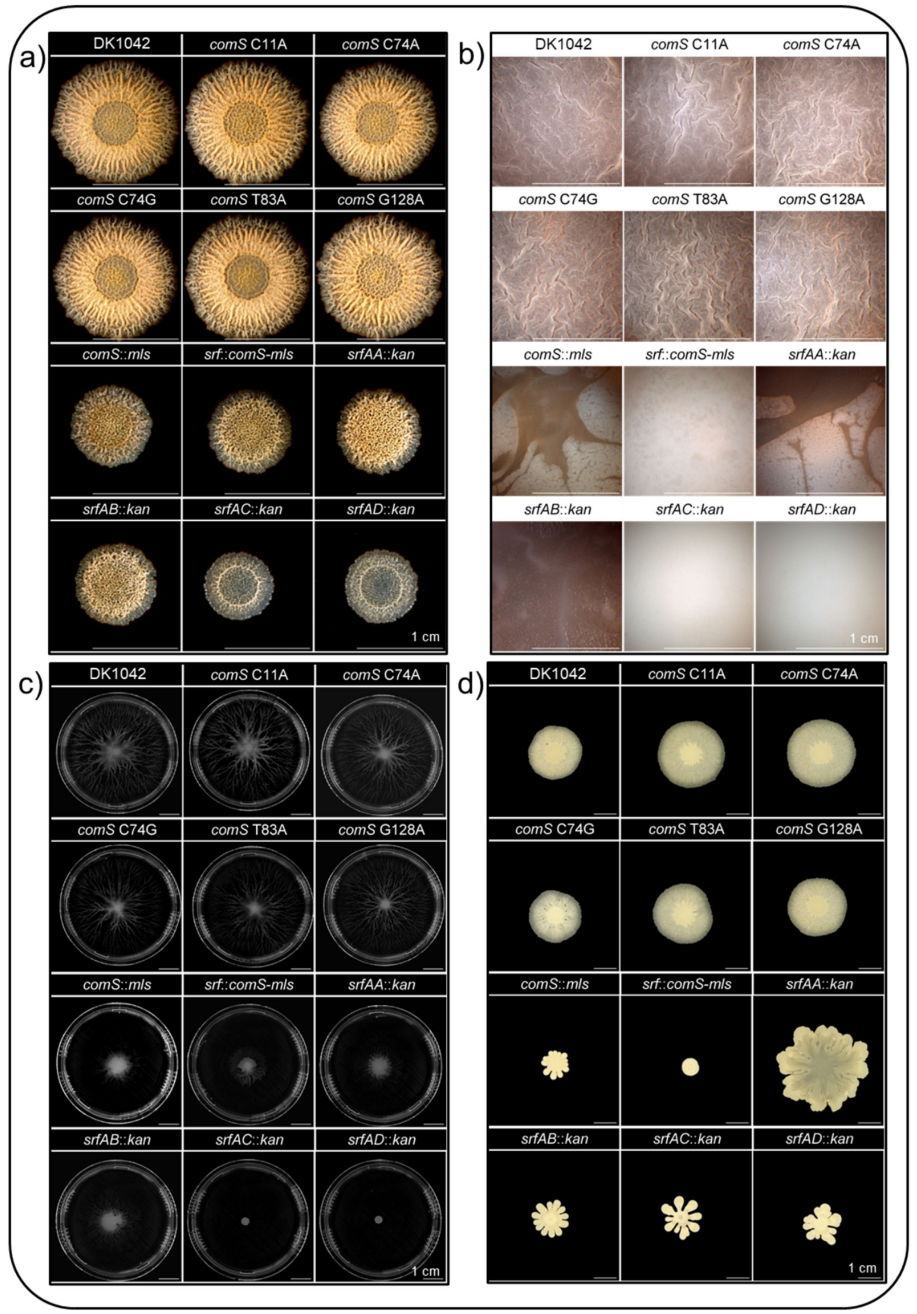
Multicellular differentiation phenotypes of *srfAABCD* and *comS* mutants under various conditions. **(a)** Biofilm formation on solid medium, highlighting differences in colony architecture **(b)** Pellicle development at the air-liquid interface, representing the ability to form floating biofilms **(c)** Sliding motility, indicating surface expansion in the absence of flagellar function, and **(d)** Swarming ability, reflecting coordinated flagellum-dependent motility across semi-solid surfaces.

The phenotypes of the premature stop codon mutations in *comS* were subsequently compared to different *srf* mutants to investigate their respective roles in multicellular differentiation, including biofilm formation, pellicle development at the air-liquid interface, sliding motility, and swarming behavior (Figure 4).

On solid biofilm-inducing medium (MSgg; Branda et al., 2001), wild type strain DK1042 formed structured, wrinkled colonies, indicative of robust biofilm development. These morphology characteristics are associated with matrix production, endospore formation, and programmed cell death, features that collectively contribute to the spatial organization and wrinkling of mature biofilms (Gingichashvili et al., 2017; Arnaouteli et al., 2021). All *comS* stop codon mutants (C11A, C74A, C74G, T83A, and G128A) retained complex colony architecture comparable to the wild type, suggesting that truncation of ComS does not impair biofilm formation at the colony level (Figure 4a). In contrast, mutants harboring disruptions in the *srf* operon (*srfAA*:*kan*, *srfAB*:*kan*, *srfAC*:*kan*, *srfAD*:*kan*) showed reduced vein-like wrinkling and limited colony expansion. Moreover, the strains exhibited diminished spatial organization, with poorly defined inner, middle and outer zones. Notably, mutants carrying a *comS* deletion, accompanied with *srfAB* disruption within the overlapping sequence (*comS*:*mls*) or expressing *comS* in the absence of *srfAABCD* (*srf*:*comS*-*mls*) displayed similar altered phenotypes. The colonies were flatter and smaller than the wild type, but retained some degree of structural organization (Figure 4a).

Interestingly, the *srfAA* deletion mutant entirely lacked inner colony structuring and exhibited disorganized wrinkling across the colony surface, deviating from the typical vein-like pattern observed in wild type biofilms. The *srfAC* and *srfAD* mutants maintained central ring-like structure, but showed minimal pigmentation, reduced colony size, and nearly absent wrinkling. Overall, while all mutations in the *srf* operon exhibited severely impaired biofilm morphology and differentiation, the introduction of targeted stop codons in *comS* had no noticeable effect on colony architecture. This confirmed that surfactin production, rather that ComS-mediated competence regulation, is critical for maintaining structured multicellular biofilm formation on solid surfaces (Figure 4a).

At the air-liquid interface, the wild type and all *comS* stop codon mutants formed robust, wrinkled pellicle with reddish-brown color (Figure 4b). This pigmentation is presumably caused by the complex formation of pulcherriminic acid and iron to the reddish-brown pulcherrimin (Yuan et al., 2020). In contrast, strains lacking genes of the *srf* operon or carrying a full *comS* deletion failed to form stable pellicles. Instead, they produced thin, fragile surface films or remaining entirely dispersed in the medium (Figure 4b). The most severe defects were observed for the *srfAC*, *srfAD* and *srf* operon deletion strains, which were completely unable to establish pellicles and exhibited a pale white color in place of the typical red-brown pigmentation seen in the wild type (Figure 4b). These observations confirm that surfactin, but not ComS, is essential for pellicle integrity, and that *comS* stop mutants maintain sufficient surfactin expression to support pellicle formation (Figure 4b).

MSggN (Fall et al., 2006) soft agar plates were employed to study sliding motility, which is a form of passive, flagella-independent surface expansion (Kearns, 2010). Under these conditions, all *comS* point mutation strains displayed colony spreading comparable to the dendritic patterns of the wild type (Figure 4c). In contrast, surfactin-deficient mutants exhibited severely impaired expansion and extreme reduction of dendritic structures, demonstrating that sliding motility depends on surfactin, but is independent of ComS. Notably, *srfAC* and *srfAD* mutants showed the strongest defect: Colonies remained compact and lacked any visible spreading (Figure 4c).

Swarming, a flagellum-dependent form of multicellular movement on semi-solid surfaces, remained unaffected in *comS* point mutation strains, which exhibited swarming patterns comparable to the wild type (Figure 4d). This behavior is characterized by circular wave-like expansion, reflecting coordinated motility. In contrast, strains carrying deletions in *srfAB*, *comS*, *srfAC*, or *srfAD* exhibited impaired swarming motility, characterized by disorganized colony morphology with irregular offshoots and uneven radial expansion. Interestingly, *srfAA* deletion resulted in increased colony expansion, yet the pattern remained disordered, indicating a similar disruption in coordinated movement. These phenotypes implicate an essential role of surfactin in facilitating collective swarming behavior. Notably, deletion of the entire *srf* operon combined with reintroduction of *comS* (*srf*:*comS*-*mls*) led to a complete loss of swarming ability, further reinforcing the requirement of surfactin for motility (Figure 4d).

**Figure 5:**
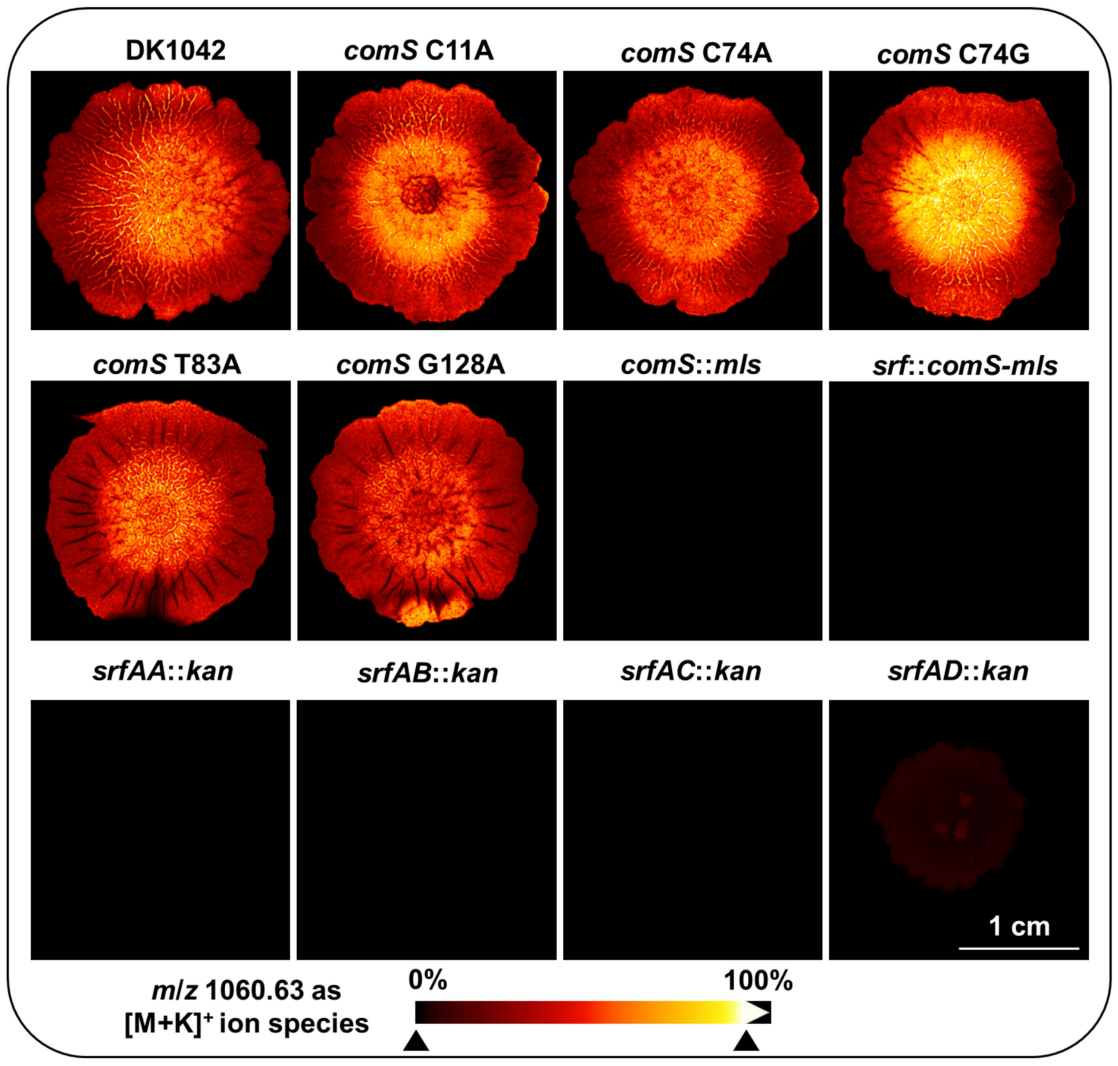
MALDI-MS images of surfactin C14 in *comS* and *srfAABCD* mutant biofilms. Representative image of surfactin production in wild type (3A38), *comS* SNP strains (C11A, C74A, C74G, T83A, and G128A), and various *srf* mutants (*srfAA:kan*, *srfAB:kan*, *srfAC:kan*, *srfAD:kan, comS:mls*, *srf:comS-mls*) was displayed. The scale bar at the lower right corner indicates 1 cm. The surfactin signal was detected at a mass to charge ratio (*m/z*) of 1060.63 as [M + K]^+^ ion species. In the heatmap representation, black indicates signal absence, while red to yellow corresponds to increasing amounts of surfactin.

All of the above phenotypic results indicate that the introduced stop codons in *comS* did not affect surfactin production. To validate this assumption, biofilms were subsequently cultivated on mixed cellulose ester membranes (Supplementary Figure S2) and analyzed by matrix-assisted laser desorption/ionization mass spectrometry imaging (MALDI-MS imaging, MALDI-MSI) (Friebel et al., 2025). Colonies grown under these conditions displayed comparable morphologies to biofilms grown on MSgg medium only (Figure 4a), suggesting that the use of mixed cellulose ester membranes does not alter biofilm architecture (Supplementary Figure S2). The MALDI-MSI technique allowed spatial visualization of surfactin distribution within the colony at a resolution of about 50 μm (with the used settings). All identified surfactin variants (C13-C15) were detected predominantly in the positive ion mode as potassium adduct ions ([M + K]^+^). For example, the three surfactins are detected at mass-to-charge ratios (*m*/*z*) of 1046.6, 1060.63, and 1074.6, respectively. In all cases this is within 12 ppm of the calculated *m*/*z* values. In Figure 5, the distribution of surfactin C14 is shown as a representative signal, however the other isoforms showed essentially identical spatial distributions in line with previous MALDI-MS imaging studies on wild type *B. subtilis* strains (Friebel et al., 2025; Brockmann et al., 2019; Si et al., 2016).

While MALDI-MSI is not a quantitative method without use of internal standards or additional quantitation of extracts, the relative signal intensities can still provide spatial insights into the localization and relative abundance of metabolites, in particular if the ion type is investigated in comparably prepared samples (Figure 5). Interestingly, surfactin appeared to accumulate more strongly in the center of the biofilm and extended into the middle zones, although the signal was detectable across the entire colony. This pattern suggests a central production or accumulation zone with broader distribution throughout the biofilm structure (Figure 5).

Consistent with previous phenotypic observations, surfactin was detected in all *comS* point mutation strains (*comS* C11A, C74A, C74G, T83A, and G128A) carrying premature stop codons, confirming that the introduced mutations did not impair *srf* operon function. In contrast, strains with deletions of *comS*, *srfAA*, *srfAB*, or *srfAC*, as well as the *srf*:*comS*-*mls* mutant, did not produce surfactin (Figure 5; Supplementary Figures S3, S4). However, the absence of surfactin, resulting in a black image, does not indicate a lack of growth, as colonies were present for all these strains (Supplementary Figure S2). Notably, surfactin was still detected, although at low levels, in the *srfAD* mutant (Figure 5). This gene encodes a type II thioesterase that acts as a proofreading enzyme to recycle misacylated PCP domains rather than directly participating in surfactin assembly. Accordingly, surfactin is still produced in the absence of SrfAD. However, the observed impairment in multicellular behavior indicates that the surfactin biosynthetic machinery may not be fully functional in the absence of *srfAD*, potentially due to reduced efficiency or accumulation of defective intermediates (Figure 5; Supplementary Figures S3, S4).

Taken together, swarming and sliding assays, biofilm and pellicle formation, as well as MALDI-MS imaging confirmed that the stop codon mutations introduced into *comS* did not disrupt *srf* operon functionality and demonstrate that it should be possible to decouple competence regulation from surfactin-mediated multicellular behavior through precise genomic modification. Next, we therefore analyzed the impact of *comS* mutations on competence development.

### 3.3 Strain-specific competence promoter activity in *Bacillus subtilis*

**Figure 6:**
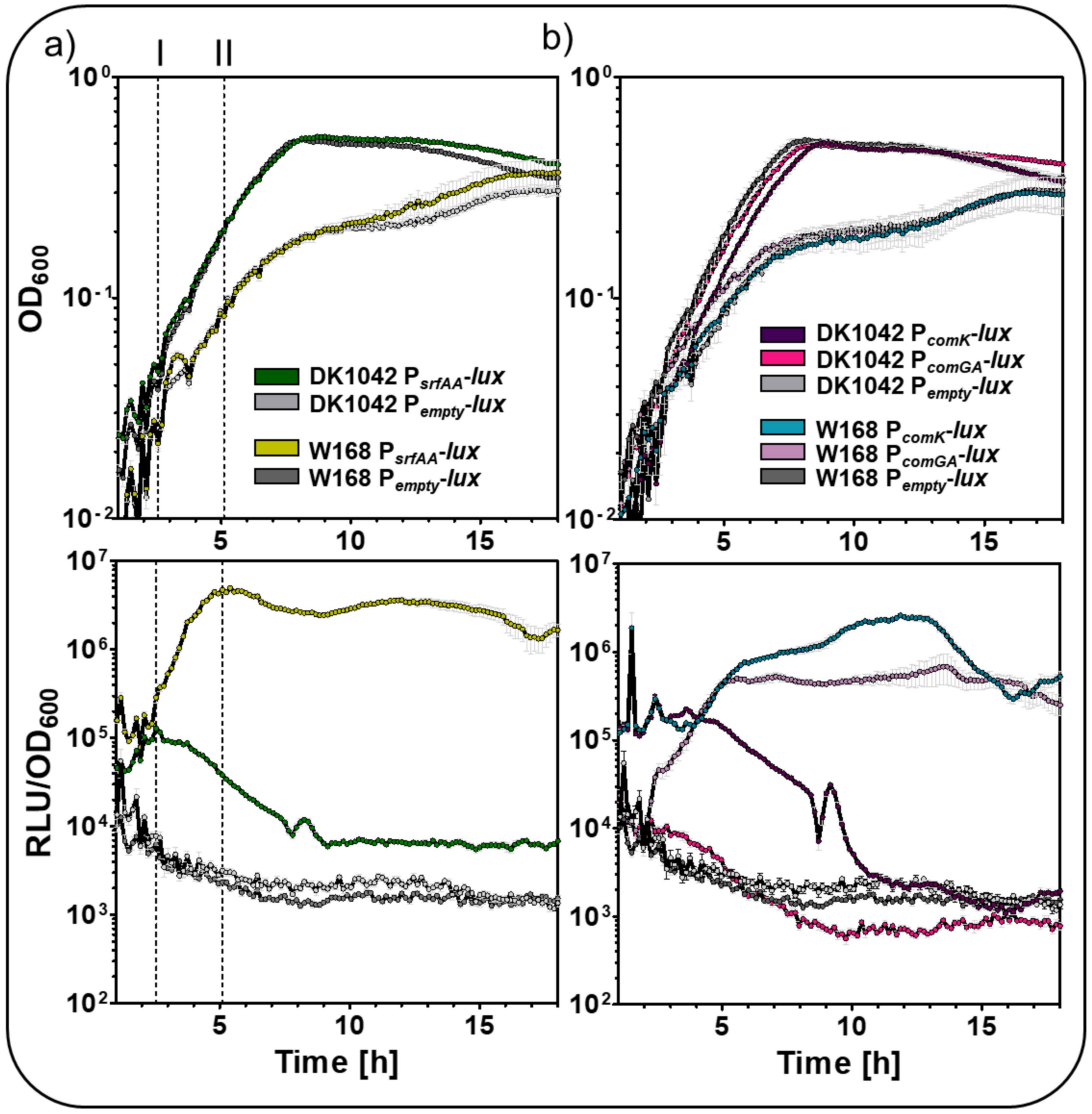
Activity of competence associated promoters in the laboratory strain W168 and biofilm forming DK1042. While the upper graphs depict the growth as function of OD_600_ over time, the lower graphs represent the relative luminescence units (RLU) normalized to the corresponding OD_600_ values over time. The reporter fusion of **(a)** *P_srfAA_*-*lux*, **(b)** *P_comK_*-*lux*, and *P_comGA_*-*lux* were applied for monitoring the dynamic and intensity of promoter activity during growth. Dashed lines indicate the maximum *P_srfAA_* activity in (I) *B. subtilis* DK1042, and (II) *B. subtilis* W168 and *P_emtyp_*-*lux* served as a negative control, indicating the background basal activity of the reporter strains. The standard deviation of the biological and technical triplicates was included as error bars to each time point of measurement.

A comparative analysis of competence-related promoter activities was conducted, focusing on expression patterns and intensities in the laboratory strain W168 and the biofilm-forming strain DK1042. Previous studies have shown that W168 exhibits approximately 1,000-fold higher transformation efficiency than DK1042 (Konkol et al., 2013; Miras and Dubnau, 2016), indicating that W168 should be more suitable for detailed investigation of competence regulation. Interestingly, the two *B. subtilis* strains displayed notable differences in growth behavior under competence-inducing conditions. When cultured in MNGE medium, strain DK1042 entered stationary phase after approximately 8 h of growth, whereas W168 grew more slowly and did not reach a stable stationary phase within the 18 h measurement period (Figure 6a).

Competence is controlled by quorum sensing-mediated activation of ComA, which initiates expression of the *srf* operon and *comS* by binding to the P*_srfAA_* promoter region (Roggiani and Dubnau, 1993). Since expression of this locus is essential for competence development, P*_srfAA_* activity was first examined in both strains (Figure 6a), which uncovered striking differences: In W168, promoter activity peaked around 5 h and then maintained an almost constitutive expression pattern (Figure 6a, II). In contrast, DK1042 showed an early peak in P*_srfAA_* activity at about 2 h, with promoter activity declining after reaching its peak, indicating a brief and tightly regulated window of surfactin and *comS* expression (Figure 6a, I). Moreover, P*_srfAA_* activity was approximately 30-fold lower in DK1042, compared to that of W168 (Supplementary Figure S5a), which is in agreement with the overall lower competence in this strain.

Since ComS has a higher binding affinity for the proteolytic complex MecA-ClpCP, it competitively inhibits ComK degradation, allowing ComK to accumulate and activate the competence regulon (D'Souza et al., 1994). P*_comK_* was therefore chosen as an indicator of competence initiation and P*_comGA_* as representative of the ComK-regulated, so-called late competence genes. Similar to the differences observed for P*_srfAA_* activity between the two strains, both P*_comK_* and P*_comGA_* activities differed significantly (Supplementary Figure S5a). In W168, P*_comK_* exhibited a relatively high basal activity compared to P*_empty_*-*lux* control and showed an approximately ten-fold increase, reaching its maximum after around 12 h of growth (Figure 6b). In contrast, DK1042 showed the maximum P*_comK_* activity already after around 4 h, followed by a decline to baseline levels by the onset of stationary phase (Figure 6b). However, the maximum was around 12-fold lower in comparison to W168. The P*_comGA_* activity in W168 mirrored the expression pattern of P*_comK_*, though it was approximately four-fold lower (Supplementary Figure S5b).

Surprisingly, no P*_comGA_* activity above the background reporter signal was detected in DJ1042, which was approx. 90-fold lower when compared to W168 (Supplementary Figure S5c). While this does not conclusively prove the absence of P*_comGA_* expression, it suggests that the activity is extremely low and below the sensitivity of the assay applied (Figure 6b).

Taken together, these results demonstrate that W168 exhibits markedly stronger and more sustained activity of competence-associated promoters (P*_srfAA_*, P*_comK_*, and P*_comGA_*) compared to DK1042, where promoter activities were transient and substantially weaker (Supplementary Figure S5, S6; Figure 6). Thus, W168 represents a more suitable strain for the analysis of competence-related gene expression and hence competence development.

### 3.4 Stop mutations in *comS* inhibit competence development

**Figure 7:**
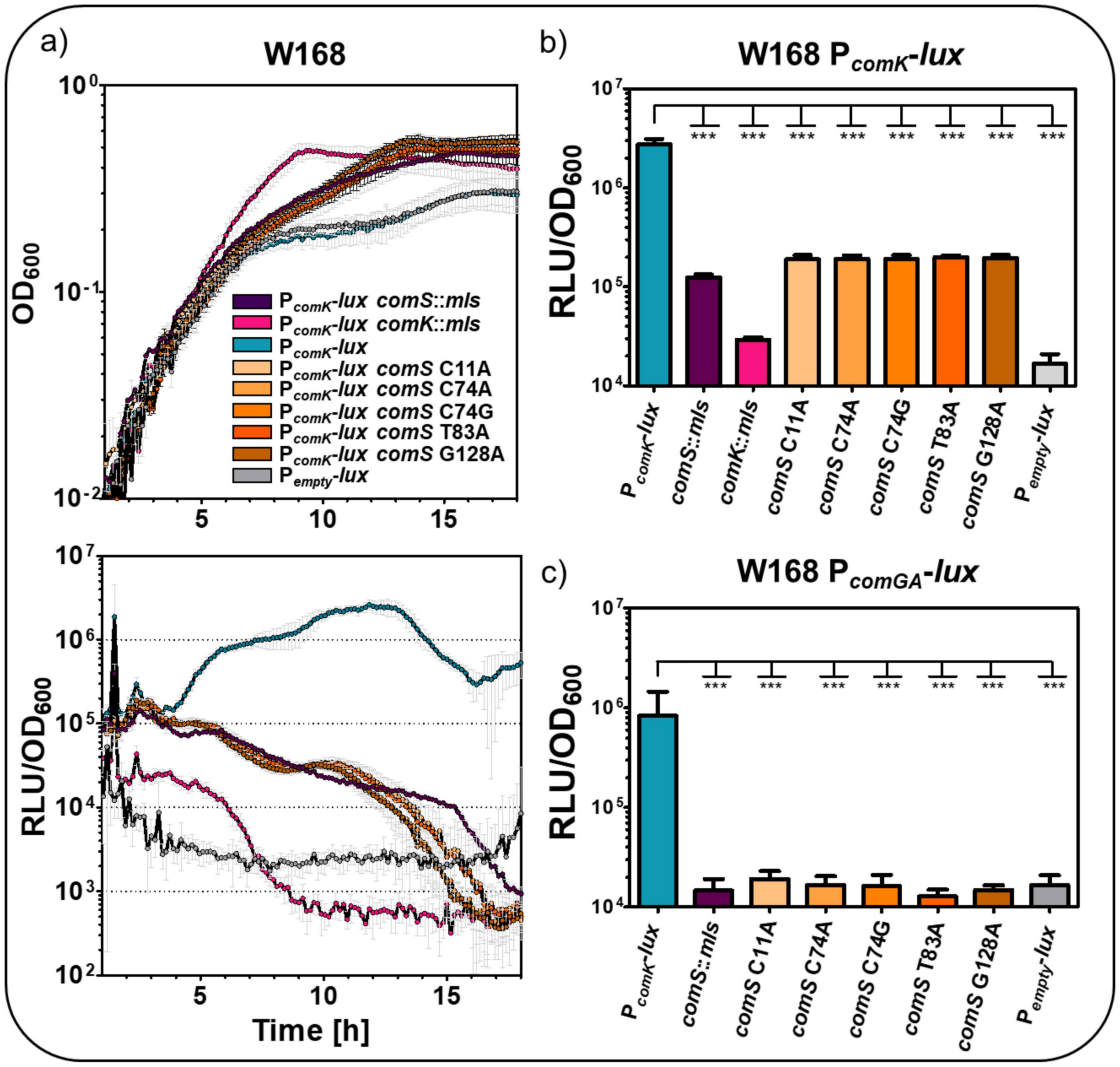
Impact of *comS* point mutations on competence development in *B. subtilis* W168. **(a)** The activity of P*_comK_*-*lux* in absence and presence of *comS* and *comK* deletion as well as the *comS* point mutations (C11A, C74A, C74G, T83A, and G128A) was monitored by measurement of relative luminescence units normalized to the corresponding OD_600_ values. P*_empty_*-*lux* served as control, indicating background activity of the reporter strain. The growth was depicted as function of OD_600_ over time. **(b)** Maximum RLU/OD_600_ values of P*_comK_*-*lux* in absence and presence of *comK* and *comS* mutations. **(c)** Maximum RLU/OD_600_ values of P*_comGA_*-*lux* in absence and presence of *comS* mutations. The standard deviation of the biological and technical triplicates was included as error bars to each time point of measurement. Statistical significance was assessed using a one-way ANOVA followed by Dunnett’s post-hoc test comparing each mutant to the wild type. Significance is indicated as follows: ns = not significant; * = *p* < 0.05; ** = *p* < 0.01; *** = *p* < 0.001.

The target-specific stop mutations in *comS* were subsequently introduced into *B. subtilis* W168 P*_comK_*-*lux* and P*_comGA_*-*lux* reporter strains to investigate the functional relevance on competence development. These constructs were compared with the wild type background as well as with *comS* and *comK* mutants. A promoterless P*_empty_-lux* strain served as a background control. In the wild type strain, P*_comK_* activity reached its maximum after approximately 12 h of growth (Figure 7a).

In contrast, *comS* mutant strains exhibited drastically reduced P*_comK_* activity, up to around 14-fold decrease relative to the wild type at the peak time point (Figure 7b). All *comS* point mutations resulted in P*_comK_* activity comparable to the *comS* deletion strain, indicating that the introduction of premature stop codons within *comS* is sufficient to disrupt competence initiation (Figure 7b). Deletion of *comK* led to even lower P*_comK_* activity, likely due to the absence of ComK-mediated positive autoregulation of its own promoter (Figures 7a,b) (Berka et al., 2002; Van Sinderen and Venema, 1994). Interestingly, *comS* and *comK* mutations also affected growth dynamics, with the mutant strains entering a stationary phase after approximately 13 h and 9 h, whereas the wild type did not reach the stationary phase during the 18 h measurement period. This growth behavior resembles that of the biofilm-forming strain DK1042 (Figure 6a). Furthermore, P*_comGA_* activity, which reflects activation of the ComK regulon, was significantly reduced in all *comS* point mutation strains, again reaching levels comparable to the *comS* deletion mutant (Figure 7c; Supplementary Figure S5d). This strongly suggests that these mutations completely disrupt the ComS-dependent activation of downstream competence genes.

Overall, these results indicate that the stop mutations in *comS* severely compromise ComS function in activating the ComK-dependent competence pathway. Even though the mutations do not alter the amino acid sequence of the overlapping *srfAB*, these mutations mirror a full *comS* deletion in terms of P*_comK_* and P*_comGA_* activity, highlighting the critical importance of *comS* integrity for competence development.

## 4 Discussion

Multicellularity represents a fundamental evolutionary adaptation strategy in bacteria, enabling populations to coordinate complex behaviors, respond adaptively to environmental changes, and divide labor across subpopulations (Branda et al., 2001; Schultz et al., 2009; Aguilar et al., 2007). In *B. subtilis*, certain matrix-producing cells differentiate into competent cells, which are capable of acquiring exogenous DNA from the environment (López et al., 2009d). This differentiation is tightly regulated by quorum sensing and the expression of the *srf* operon, which encodes both ComS and the heptalipopeptide surfactin (Cosmina et al., 1993; D'Souza et al., 1994). While ComS facilitates competence development, surfactin drives multicellular motility behaviors such as swarming, sliding, and exhibits antimicrobial activity (D'Souza et al., 1994; Kinsinger et al., 2003). Despite being co-transcribed within the *srf* operon, the functional separation between ComS and surfactin has been poorly explored due to overlapping coding regions.

In this study, five different stop mutations were introduced into *comS*, which successfully abolished competence while preserving surfactin production and associated multicellular behaviors such as biofilm formation, swarming, and sliding motility (Figures 4, 7). Importantly, this point mutation-based approach maintained the structural and regulatory integrity of the overlapping *srfAB* coding region, demonstrating the feasibility of precise operon-embedded gene editing. Our results demonstrate that competence and multicellular differentiation, although genetically linked, can be functionally separated. Surfactin production alone proved sufficient to sustain surface-associated motility and structural organization in the absence of ComS, while the latter exclusively affected ComK-dependent gene expression and hence competence development (Figures 6, 7).

Further dissection of the *srf* operon revealed surprising phenotypic differences between the deletions of its individual components. While mutants lacking *srfAA*, *srfAB*, or *srfAC* are incapable of producing surfactin and show compromised biofilm development, the *srfAD* mutant, which retained surfactin biosynthesis, nonetheless exhibited severe defects in biofilm and pellicle formation

(Figure 4). The *srf* operon encodes a modular NRPS assembly line responsible for the biosynthesis of surfactin (Vater et al., 1997). Within this system, SrfAA, SrfAB, and SrfAC coordinate the stepwise assembly and cyclisation of surfactin, while SrfAD functions as a proofreading thioesterase, recycling misacylated PCP domains (Vater et al., 1997; Koglin et al., 2008; Yeh et al., 2004). If uncorrected, these misacylations can obstruct the essential 4’-PP thiol group. Activation of PCP domains further requires the phosphopantetheinyl transferase Sfp, which is absent in standard laboratory strains such as *B. subtilis* W168 (Cosmina et al., 1993; Quadri et al., 1998). Deletions of *srfAC* and *srfAD* had the most disruptive impact on biofilm and pellicle formation (Figure 4). While SrfAC catalysis final peptide cyclization and termination, it also aids substrate channeling during synthesis. SrfAD, in turn, contributes both to quality control and to initiating synthesis via fatty acid transfer to the first module of SrfAA (Yeh et al., 2004; Steller et al., 2004). Disrupting these functions might result in the accumulation of stalled or toxic intermediates, potentially perturbing membranes, inducing metabolic imbalances, or producing non-functional surfactin variants.

**Figure 8:**
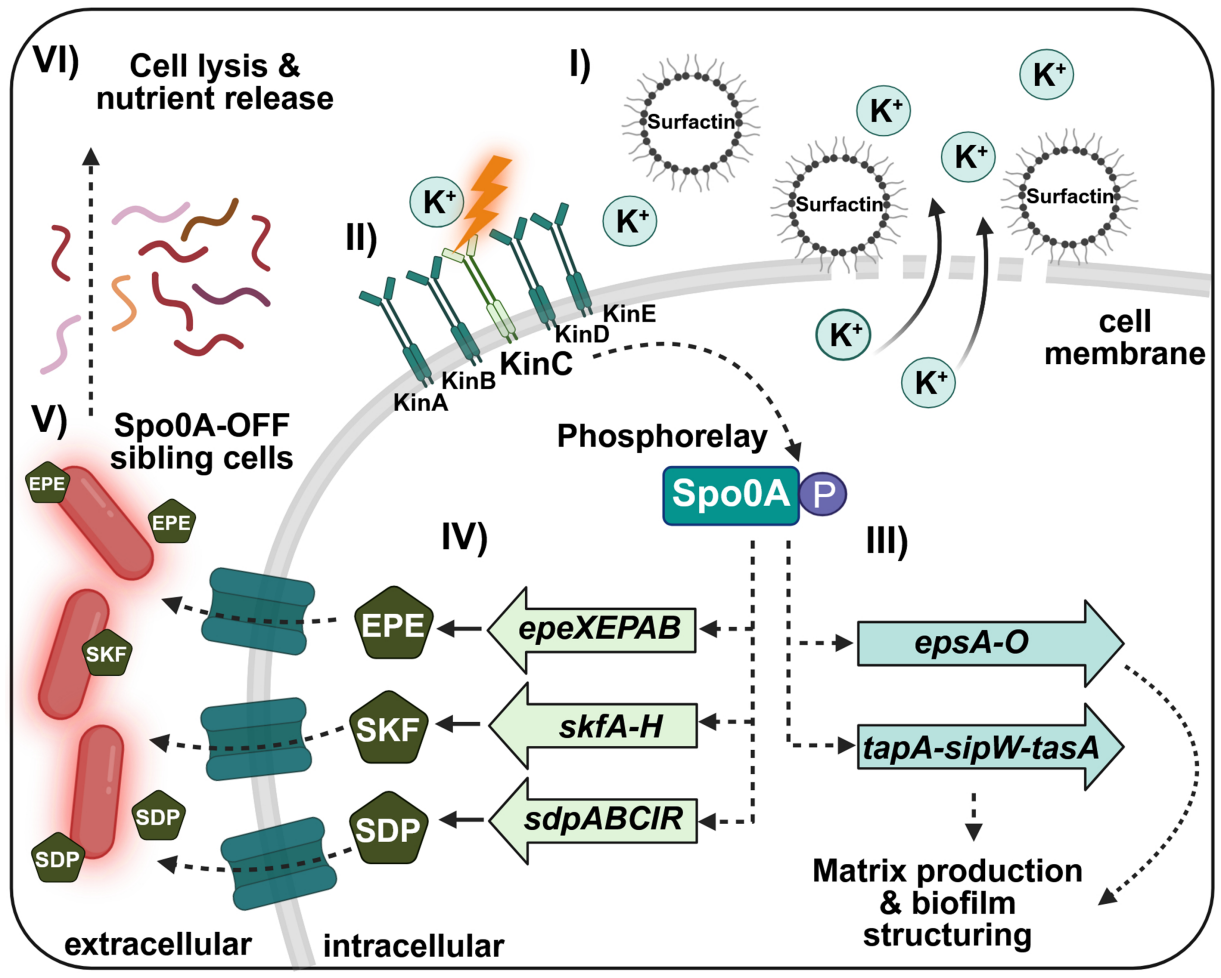
Surfactin-mediated quorum sensing and differentiation. (I) Surfactin acts as a quorum sensing signal by forming pores in the cell membrane, leading to potassium ion leakage and reduced intracellular potassium levels. (II) This ionic imbalance is detected by the histidine kinase KinC, which initiates the phosphorelay ultimately resulting in the activation of Spo0A (Spo0A ~ P). (III) At low Spo0A ~ P levels, a subpopulation of cells differentiates into matric producers, enabling transcription of the *epsA-O* and *tapA-sipW-tasA* operons required for matrix production and biofilm structuring. (IV) Moreover, low Spo0A ~ P levels activate expression of the *epeXEPAB*, *skfA-H*, and *sdpABCIR* operons, encoding the cannibalism toxins EPE, SKF, and SDP, respectively. (V) These toxins selectively lyse sibling cells that have not yet initiated sporulation (Spo0A-OFF), (VI) thereby releasing nutrients that are recycled by the producing subpopulation, ensuring their survival.

Surfactin plays a pivotal role in motility. Swarming, a flagella-dependent process, and sliding, a flagella-independent form of surface expansion, are both facilitated by its ability to reduce surface tension. Deletion of any *srf* gene markedly impaired sliding, particularly in *srfAC* and *srfAD* mutants (Figure 4). Swarming was also compromised, although not abolished. The inability of functional flagella to compensate for the absence of surfactin underscores its unique biophysical function (Kearns and Losick, 2003; Senesi et al., 2004; Ghelardi et al., 2012). Interestingly, the *srfAA* deletion resulted in irregular but enhanced colony expansion, possibly due to accumulation of non-functional surfactin intermediates (Figure 4d). These findings reflect both the specific role of surfactin in reducing surface tension, as well as its contribution to the broader regulatory and structural framework that governs multicellular behavior (Kinsinger et al., 2003; Angelini et al., 2009): Beyond its biophysical effects, surfactin also functions as a quorum sensing molecule. By inducing potassium ion leakage, it lowers intracellular potassium levels (Figure 8, I). This ionic shift is sensed by the histidine kinase KinC, which triggers a phosphorylation cascade culminating in Spo0A activation (Spo0A ~ P) (Figure 8, II; López et al., 2009a; Lopez et al., 2009b).

At low levels of Spo0A ~ P, distinct subpopulations differentiate into matrix producers through SinI-mediated inhibition of SinR, enabling expression of the *epsA-O* and *tapA-sipW-tasA* operons required for matrix production (Figure 8, III; Kearns et al., 2005; Fujita et al., 2005). Simultaneously, low Spo0A ~ P levels induce expression of the *skfA*-*H*, *sdpABCIR*, and *epeXEPAB* operons, which encode the cannibalism toxins sporulation killing factor (SKF), sporulation delay protein (SDP), and epipeptide (EPE), respectively (Figure 8, IV; Fujita et al., 2005; González-Pastor et al., 2003; Popp et al., 2021). These toxins selectively lyse sibling cells that have not yet initiated sporulation, thereby releasing nutrients that benefit the producing cells (Figure 8, V-VI; González-Pastor, 2011). While surfactin and SKF appear to be distributed throughout the entire colony, EPE localizes predominantly to the outer edges, whereas SDP is concentrated toward the colony center (Supplementary Figures S7–S9; Friebel et al., 2025). Importantly, both surfactin and cannibalism toxins disrupt membranes, leading to cell lysis and nutrient release, suggesting a shared role in resource acquisition and biofilm structuring (Friebel et al., 2025; López et al., 2009d). In the absence of surfactin, these differentiation processes, including matrix production and toxin expression, are impaired (López et al., 2009c; López et al., 2009d). Notably, it was previously shown that SKF production is specifically abolished in a *srfAC* mutant (Supplementary Figure S7; Dinesen et al., 2025), which resembles the observation that EPE and also SDP are barely detectable in a *srfAC* deletion strain, while its production remains unaffected in other *srf* mutants (Supplementary Figure S8, S9; Dinesen et al., 2025). These findings suggest that SrfAC plays a critical role in regulating toxin biosynthesis, but the underlying mechanism remains unclear.

The integration of surfactin biosynthesis within the competence regulatory network adds another layer of functional significance. Since surfactin and *comS* are co-transcribed from the *srf* operon, the coupling of antimicrobial compound production with competence development may represent an adaptive strategy to balance genetic diversification and community-level survival. Cell lysis mediated by surfactin, as well as the cannibalism toxins SKF, SDP and EPE presumably facilitates DNA release, which in turn provides substrates for uptake by competent cells. This dual role of surfactin is further reflected by its regulation via two quorum sensing systems: the ComQXPA circuit, which governs competence initiation, and the broader Rap-Phr signaling network, which modulates the phosphorylation states of ComA, DegU, and Spo0F (Ng and Bassler, 2009; Miller and Bassler, 2001). These cell–cell communication systems enable the community to assess population density and coordinate multicellular behaviors in a synchronized and community optimized manner, which creates a phenotypic heterogeneity within genetically identical populations (Miller and Bassler, 2001; Gallegos-Monterrosa et al., 2021). Thus, surfactin production is not only functionally but also regulatory embedded within the multicellular differentiation and the competence pathway.

Experimental observations using the *B. subtilis* strain W168 supported the existence of regulatory interdependence between competence development and growth dynamics (Figures 6, 7). W168 is naturally highly competent, predominantly due to the absence of the plasmid-borne *comI* gene and an inactivating point mutation in the promoter region of *degQ* (Konkol et al., 2013). This mutation results in reduced *degQ* expression and consequently lower levels of DegU~P, which in turn leads to de-repression of the *srf* operon, facilitating the expression of *comS*, and subsequently stabilization of ComK (Miras and Dubnau, 2016; Yang et al., 1986). Deletion of either *comS* or *comK* in this background resulted in a growth phenotype similar to that of the low competent strain DK1042, characterized by an earlier onset of stationary phase (Figures 6, 7). These findings suggest that competence development may contribute to extending the exponential growth phase, possibly through stress-responsive or metabolic adaptations that enhance cellular fitness under nutrient-limited conditions.

*Bacillus subtilis* encounters a wide range of stressors in its natural habitat, including carbon, nitrogen, phosphate, and sulfur limitation, as well as oxidative and osmotic stress (Hecker et al., 2007). In such stress conditions, the division of labor becomes crucial, triggering the initiation of diverse differentiation strategies (Vlamakis et al., 2008). These adaptive responses are governed by the activity of Spo0A and quorum sensing mechanisms that assess population density, which orchestrate cell fate decisions in response to nutrient stress (Vlamakis et al., 2008). While at low levels of Spo0A ~ P individual cells may differentiate into competent cells, or engage in cannibalistic behavior, high levels of Spo0A ~ P determine the final commitment to sporulation (Schultz et al., 2009). This results in the formation of highly resilient endospores that can survive extreme environmental conditions, such as desiccation, ultra violet (UV) radiation, and acidic/alkaline pH values. Despite their exceptional resistance and long-term survival capability, endospores are metabolically dormant and thus incapable of growth, replication, or direct competition with actively dividing bacteria for nutrients and position within their ecological niche (Checinska et al., 2015). Moreover, endospore formation is highly energy-consuming and irreversible, making it a last-resort survival strategy when all other options for adaptation have been exhausted (Parker et al., 1996).

In contrast, competence provides a more flexible and responsive adaptation strategy. The coordinated induction of competence and surfactin production allows cells to take up extracellular DNA, gain genetic diversity, overcome nutrient limitations, and maintain growth and competitiveness within their ecological niche (Schultz et al., 2009; Finkel and Kolter, 2001; Claverys et al., 2006). Surfactin may contribute to this process both directly, by promoting DNA and nutrient release through pore formation, and indirectly, by stimulating the production of cannibalism toxins (Figure 8; López et al., 2009a; Carrillo et al., 2003). This regulatory coupling enhances adaptability under fluctuating conditions and enables populations to balance survival with genetic diversification. Hence, the genetic linkage between competence and surfactin production represents an evolved strategy that maximizes both survival and evolutionary potential in heterogeneous environments. Although transformation efficiency was not assessed in this study, it would be an interesting aspect for future experiments to directly evaluate how these mutations affect competence behavior in both DK1042 and W168.

Altogether, the findings demonstrate that precise genetic editing of operon-embedded genes enables functional dissection of overlapping regulatory networks in *B. subtilis*. By separating competence from surfactin-associated multicellular behaviors, it was demonstrated that surfactin alone drives structural organization, motility, and differentiation in biofilm communities, while ComS alone affects competence development. These results underscore the role of surfactin in coordinating population traits such as antimicrobial defense, differentiation, and genetic adaptation.

From an applied perspective, the decoupling of surfactin production from competence development offers promising opportunities in biotechnology and synthetic biology. Surfactin is a high-value biosurfactant with wide-ranging applications in bioremediation, agriculture, and pharmaceuticals due to its antimicrobial, antiviral, and surface-active properties (Yuan et al., 2018; Yadav and Eswari, 2022; Bais et al., 2004). Engineering strains that efficiently produce surfactin without activating energy-consuming processes like competence or sporulation might reduce metabolic burden, thereby enhancing process efficiency and improving product yield (Klausmann et al., 2021; Guo et al., 2024). Veening et al. (2008a) and Veening et al. (2008b) showed that sporulation can divert cellular resources away from growth and other metabolic activities, reducing overall productivity. This bet-hedging strategy allows a subpopulation to survive extreme stress while others maintain growth, yet it imposes a trade-off by limiting resources available for metabolite synthesis (Veening et al., 2008b). Minimizing such energy-intensive differentiation processes could therefore free resources for products like surfactin. By decoupling surfactin production from other costly pathways such as competence and sporulation, engineered strains are expected to maintain higher growth rates and more efficient allocation of resources toward the desired product (Klausmann et al., 2021; Veening et al., 2008a). Although we did not directly quantify surfactin yield or metabolic burden in the present study, future work systematically comparing engineered strains with wild type and competence-proficient backgrounds will be important to evaluate potential trade-offs, unintended effects, and improvements in process efficiency. Such data will provide a solid basis for rational strategies to optimize *B. subtilis* strains for industrial applications, whereby targeted point mutation-based modulation provides a fine-tuned strategy for markerless strain optimization.

## Data Availability

The raw data presented in this study are publicly available here: 10.6084/m9.figshare.31092670.
