# Supplementary material for: Dissecting the role of comS-independent srf expression on multicellular differentiation and competence development in Bacillus subtilis
Source: Front Microbiol. 2026 Feb 4;17:1753310. doi: 10.3389/fmicb.2026.1753310 (PMC12915044; doi:10.3389/fmicb.2026.1753310)
Supplement: Supplementary file 1 [file Data_Sheet_1.pdf]

Supplementary Material

1 Supplementary Data

2 Supplementary Figures and Tables

Table S1: Strains applied in this study.

Strain	Genotype*	Reference
DK1042	*B. subtilis* NCIB3610 *comI* Q12L	Konkol et al., 2013 [2]
TMB5875	W168 P_{comK}-*lux_cat*::*sacA*	This study
TMB6374	W168 P_{srfAA}-*lux_cat*::*sacA*	This study
TMB6382	DK1042 P_{srfAA}-*lux_cat*::*sacA*	This study
TMB6537	DK1042 *srfAA*::*kan*	This study
TMB6788	DK1042 *comS*::*mls*	This study
TMB6824	DK1042 *comS* T83A	This study
TMB6825	DK1042 *comS* C74G	This study
TMB6826	DK1042 *comS* C74A	This study
TMB6858	DK1042 *comS* C11A	This study
TMB6859	DK1042 *comS* G128A	This study
TMB6862	DK1042 *srfAB*::*kan*	This study
TMB6863	DK1042 *srfAC*::*kan*	This study
TMB6864	DK1042 *srfAD*::*kan*	This study
TMB6866	DK1042 Δ*srf*::*comS*	This study
TMB6921	DK1042 *comS* C11A P_{comK}-*lux_cat*::*sacA*	This study
TMB6922	DK1042 *comS* C74A P_{comK}-*lux_cat*::*sacA*	This study
TMB6923	DK1042 *comS* C74G P_{comK}-*lux_cat*::*sacA*	This study
TMB6924	DK1042 *comS* T83A P_{comK}-*lux_cat*::*sacA*	This study
TMB6925	DK1042 *comS* G128A P_{comK}-*lux_cat*::*sacA*	This study
TMB6926	DK1042 *comS*::*mls* P_{comK}-*lux_cat*::*sacA*	This study
TMB6927	DK1042 P_{comK}-*lux_cat*::*sacA*	This study
TMB6950	DK1042 *comK*::*mls* P_{comK}-*lux_cat*::*sacA*	This study
TMB6951	DK1042 *comS* C11A P_{comGA}-*lux_cat*::*sacA*	This study
TMB6952	DK1042 *comS* C74A P_{comGA}-*lux_cat*::*sacA*	This study
TMB6953	DK1042 *comS* C74G P_{comGA}-*lux_cat*::*sacA*	This study
TMB6954	DK1042 *comS* T83A P_{comGA}-*lux_cat*::*sacA*	This study
TMB6955	DK1042 *comS* G128A P_{comGA}-*lux_cat*::*sacA*	This study
TMB6956	DK1042 *comS*::*mls* P_{comGA}-*lux_cat*::*sacA*	This study
TMB6957	DK1042 P_{comGA}-*lux_cat*::*sacA*	This study
TMB6958	W168 P_{comGA}-*lux_cat*::*sacA*	This study
TMB6959	W168 *comK*::*mls* P_{comK}-*lux_cat*::*sacA*	This study
TMB6960	W168 *comS*::*mls* P_{comK}-*lux_cat*::*sacA*	This study
TMB6974	W168 *comS*::*mls* P_{comGA}-*lux_cat*::*sacA*	This study
TMB6997	W168 *comS* C11A P_{comK}-*lux_cat*::*sacA*	This study
TMB6998	W168 *comS* C74A P_{comK}-*lux_cat*::*sacA*	This study
TMB6999	W168 *comS* C74G P_{comK}-*lux_cat*::*sacA*	This study
TMB7000	W168 *comS* T83A P_{comK}-*lux_cat*::*sacA*	This study
TMB7001	W168 *comS* G128A P_{comK}-*lux_cat*::*sacA*	This study
TMB7024	W168 *comS* C11A P_{comGA}-*lux_cat*::*sacA*	This study

TMB7025	W168 *comS* C74A P_{comGA}-*lux_cat*::*sacA*	This study
TMB7026	W168 *comS* C74G P_{comGA}-*lux_cat*::*sacA*	This study
TMB7027	W168 *comS* T83A P_{comGA}-*lux_cat*::*sacA*	This study
TMB7028	W168 *comS* G128A P_{comGA}-*lux_cat*::*sacA*	This study
W168	*B. subtilis* laboratory wild type strain	Laboratory stock

**cat*: chloramphenicol resistance; *kan*: kanamycin resistance; *mls*: macrolide, lincosamide, and streptogramine resistance

Table S2: Plasmids applied in this study.

Plasmid	Genotype*	Reference
pJOE8999	CRISPR-Cas9 vector, *kan*	Altenbuchner, 2016 [30]
pBS3C-*lux*	*sacA*' … '*sacA*, *luxABCDE*, *bla*, *cat*	Radeck et al., 2013 [71]
pJOE8999_ gRNA	pJOE8999 derivate, *kan*, gRNA targeting *srfAB*	This study
pJOE8999_gRNA_*comS* C11A	pJOE8999_ gRNA derivate, integration template for generation of *comS* C11A	This study
pJOE8999_gRNA_*comS* C74A	pJOE8999_ gRNA derivate, integration template for generation of *comS* C74A	This study
pJOE8999_gRNA_*comS* C74G	pJOE8999_ gRNA derivate, integration template for generation of *comS* C74G	This study
pJOE8999_gRNA_*comS* T83A	pJOE8999_ gRNA derivate, integration template for generation of *comS* T83A	This study
pJOE8999_gRNA_*comS* G128A	pJOE8999_ gRNA derivate, integration template for generation of *comS* G128A	This study
pBS3C*lux*_P_{comK}	*sacA*' P_{comK}-*luxABCDE_cat* '*sacA*	This study
pBS3C*lux*_P_{srfAA}	*sacA*' P_{srfAA}-*luxABCDE_cat* '*sacA*	This study
pBS3C*lux*_P_{comGA}	*sacA*' P_{comGA}-*luxABCDE_cat* '*sacA*	This study

**cat*: chloramphenicol resistance; *kan*: kanamycin resistance; *bla*: ampicillin resistance

Table S3: Oligonucleotides used in this study.

Oligonucleotide	Sequence 5'-3'	Source/Name*
TM7965	ACGGTCTCAtcgaCTGCATTCCTGCGTGAGCAAAG	*comS* upstream all point mutations fwd
TM7966	ACGGTCTCActagCAATCAGCTCCTCAAGCGGATAG	*comS* downstream all point mutation rev
TM7967	ACGGTCTCAttatCGGTTCAAACGTCTGCTCCTC	*comS* upstream C11A rev
TM7968	ACGGTCTCAataaGGCAAGCATCTTATCAGCAGC	PAM exchange C11A fwd
TM7969	ACGGTCTCAttagGATATACATTCTCCGCTGGGCC	*comS* upstream C74A rev
TM7970	ACGGTCTCActaaATCAGCTTGGACAAGCAAACAC	PAM exchange C74A fwd
TM7971	ACGGTCTCAtcagGATATACATTCTCCGCTGGGCC	*comS* upstream C74G rev
TM7972	ACGGTCTCActgaATCAGCTTGGACAAGCAAACAC	PAM exchange C74G fwd
TM7973	ACGGTCTCAcctaGCTGATTGAGGATATACATTCTCCGC	*comS* upstream T83A rev
TM7974	ACGGTCTCAtaggACAAGCAAACACAAGCTACAACGTC	PAM exchange T83A fwd

TM7975	ACGGTCTCActagCAGAAGTACAGCGGGGACG TTG	*comS* upstream G128A rev
TM7976	ACGGTCTCActagAGGGAGAAGTAGATAAAGA CCGGC	PAM exchange G128A fwd
TM7977	CACCTGATTCAAATGGACAGCCTG	*comS* check fwd
TM7978	GCATATCAATGAGCAGCAGGTGG	*comS* check rev
TM7979	GCACCGTATTCCTCCTTTGC	pJOE8999 backbone fwd
TM7980	aaacCCGTACATCGTTTGACATGA	gRNA *srfAB* fwd
TM7981	tacgTCATGTCAAACGATGTACGG	gRNA *srfAB* rev
TM7982	ACGGTCTCAagtaTTTCGTGCCGGTTGATTAAT TGCTG	PAM exchange all point mutations rev
TM7983	ACGGTCTCAtactCCGTACATCGTTTGACATGA TCG	*comS* downstream all point mutation fwd
TM7367	GAGGTCTAATGCAAGCTGTAATTC	*srfAA* check fwd
TM7368	ATCTCATAGAGCGGCACGTAATC	*srfAA* check rev
TM7369	AGATCACTTGCTTCTTGCCATTC	*srfAB* check fwd
TM7379	TTCTGCTTGTATCCCTTGTAAGC	*srfAB* check rev
TM8027	AGATGCTGAGAAGCCAGATTAAG	*srfAC* check fwd
TM8028	TTTCTGACACTGAAACGGTCG	*srfAC* check rev
TM8025	AACCTTTACCTTCTTGGATGAGC	*srfAD* check fwd
TM8026	GTGTAACTGAGCCCGAATAACAG	*srfAD* check rev
TM8067	agtctctagaCACCAGACATATCAGGGTGAC	P_{comGA} fwd
TM8068	agtcgtcgacGATTCCCTCTCCTTTCAACG	P_{comGA} rev
TM7811	cgcgaattcAGTATATGGATAACGGTCGA	P_{comK} fwd
TM7812	atgctagcGCCTCCATCCTTTTTCTGCA	P_{comK} rev
TM7512	agtcactagtGTGCAACGCATTTTCTCTTTC	P_{srfAA} fwd
TM7514	agtcggtctcaaattTCTTGAAGCCATGTATGAGTG	P_{srfAA} rev

*rev: reverse; fwd: forward

Table S4: Codon usage of *B. subtilis* before and after the introduction of point mutations (Nakamura et al., 2000).

**Point mutation**	***comS* C11A**	***comS* C74A**	***comS* C74G**	***comS* T83A**	***comS* G128A**
Old codon usage*	27.2 (ATC)	10.7 (CTC)	10.7 (CTC)	21.8 (CTT)	23.0 (CTG)
New codon usage*	9.8 (ATA)	23.0 (CTG)	4.9 (CTA)	4.9 (CTA)	4.9 (CTA)
Amino acid of *srfAB*	Isoleucin	Leucin	Leucin	Leucin	Leucin

* Codon usage as frequency per thousand

2.1 Supplementary Figures

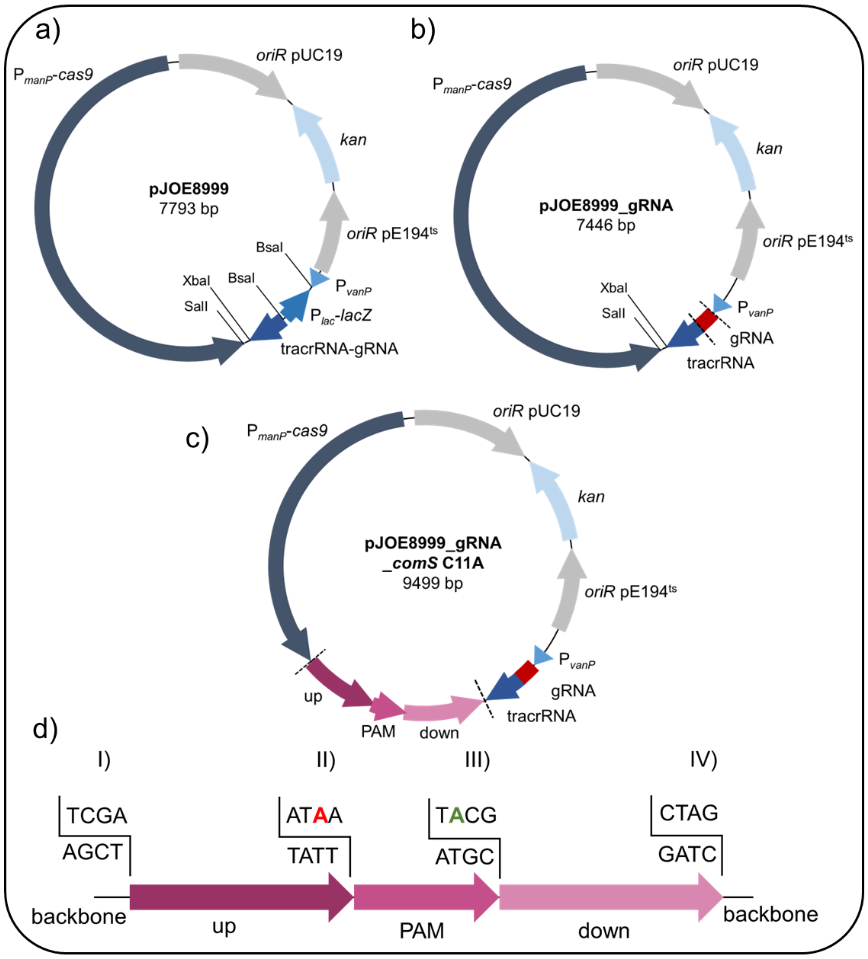

Figure S1. Cloning procedure for the generation of targeted stop mutations in *comS*.

a) The plasmid pJOE8999 was used as the CRISPR-Cas9 editing vector. It harbours a codon-optimised cas9 gene under the control of a mannose-inducible promoter (P_{manP}), a customizable guide RNA (gRNA) expression module driven by the semi-synthetic P_{vanP} promoter, a kanamycin resistance gene for selection, and dual origins of replication: *oriR* (from pUC19) for replication in *E. coli* and pE194ts for temperature-sensitive replication in *B. subtilis*. **b)** The specific gRNA targeting the *srfAB* region was cloned into the BsaI restriction sites directly upstream of the P_{vanP} promoter using Golden Gate Assembly. **c)** A homology-directed repair (HDR) template was designed to include upstream and downstream homologous regions flanking the target site in *comS*, along with a modified PAM sequence and the desired stop-gain SNP. This repair fragment was cloned into the unique XbaI and SalI sites of pJOE8999. **d)** Golden Gate Assembly was employed to ensure precise and seamless integration of all fragments using BsaI, a type IIS restriction enzyme that generates custom four-nucleotide overhangs. These overhangs facilitated both correct in-frame assembly and the introduction of SNPs within the PAM site and at the target codon in *comS*. The *comS* C11A mutation is shown as a representative example, in which the SNP introduces a premature stop codon without altering the amino acid sequence of the overlapping *srfAB* gene.

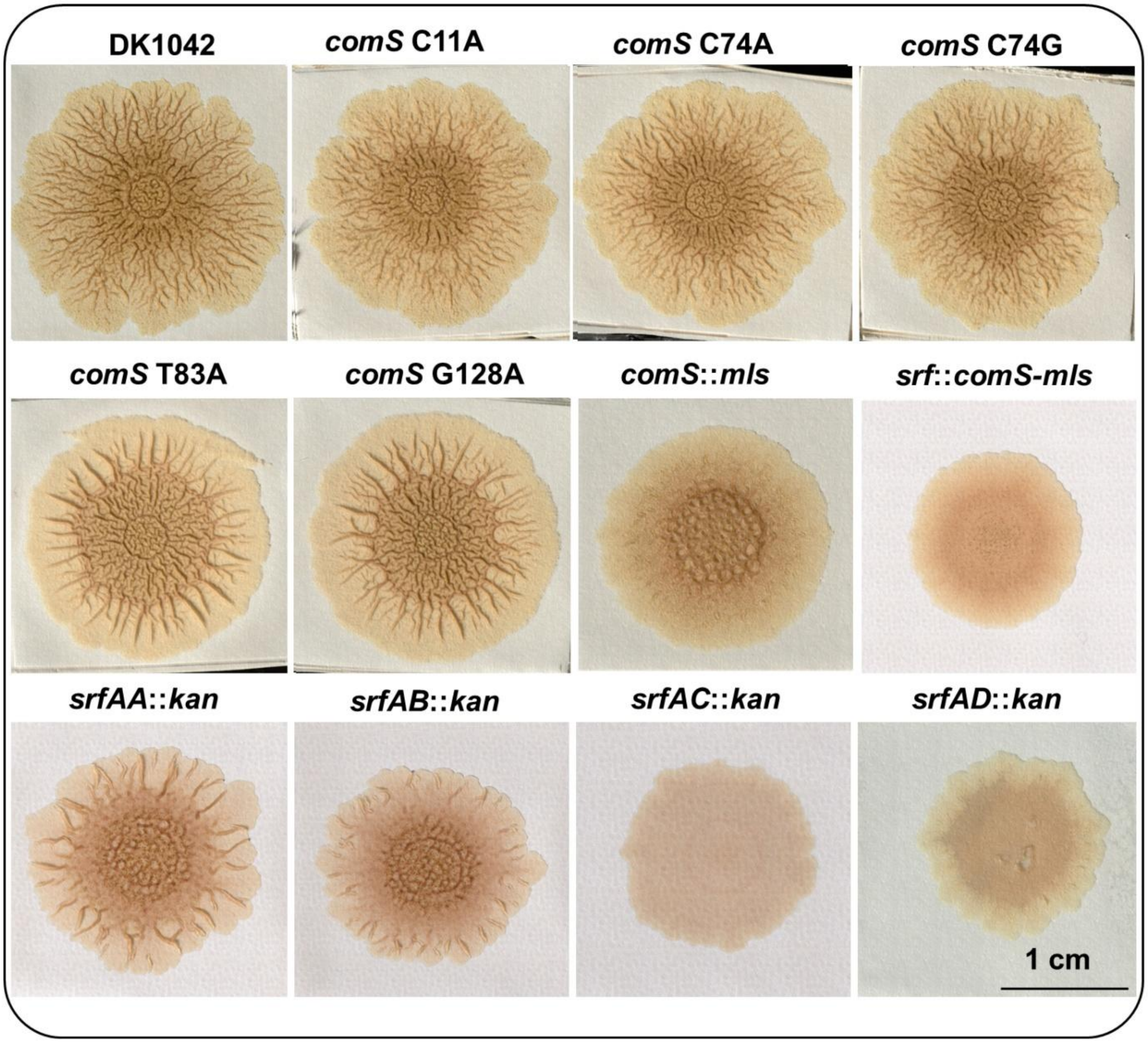

Figure S2: Biofilm formation of *comS* and *srf* operon mutants on mixed cellulose ester membranes.

Mixed cellulose ester membranes with 0.22 µm average pore size were used as subtrate to grow biofilms on solid MSgg medium for 7 days. For microscope image visualisation, OlyVia (version 3.4.1, Evident, Hamburg, Germany) was used. The wild type DK1042 was applied as control. Following scanning microscopy, the same biofilms were coated with 2,5-DHAP MALDI matrix and subjected to the MSI analysis at 50 µm pixel size.

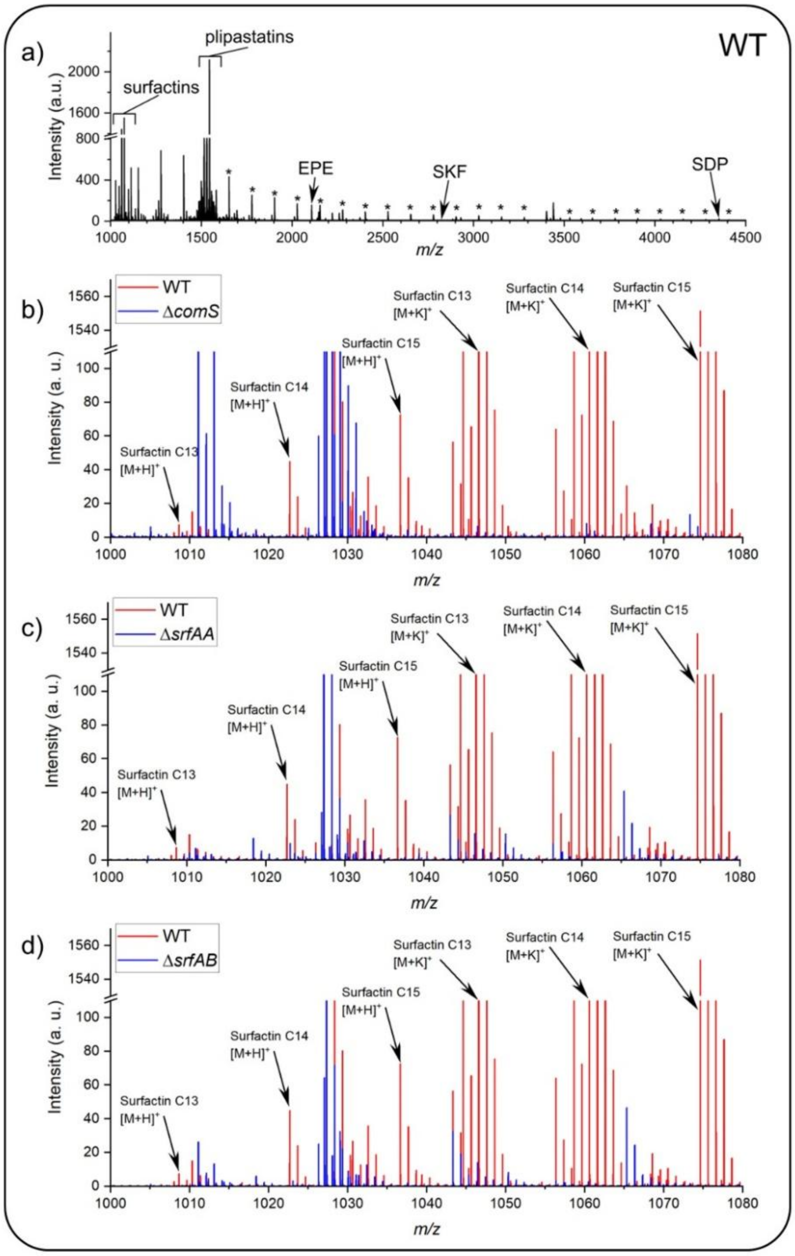

Figure S3: Summed MALDI-MS imaging spectra of *comS*, *srfAA*, *srfAB* deletion strains, and wild type DK1042.

MALDI-MS spectra of wild type DK1042 (wt; red trace), and corresponding mutants (blue). Distinct ion adducts, including $[M+H]^+$, and $[M+K]^+$ species of the respective compounds are indicated with arrows. **a)** The overview spectrum registered from wt biofilm shows the group of detected surfactins and pliplastatins (not evaluated). In addition, signals corresponding to the cannibalism toxins EPE, SDP, and SKF are recorded (see Friebel et al., 2025 for more details). Signals marked with an asterisk belong to a cyanoacrylate polymer from the used superglue. **(b-d)** Spectra from *comS*, *srfAA*, and *srfAD* deletion strains, respectively, reveal the complete loss of surfactin signals, confirming the absence of surfactin production in these backgrounds.

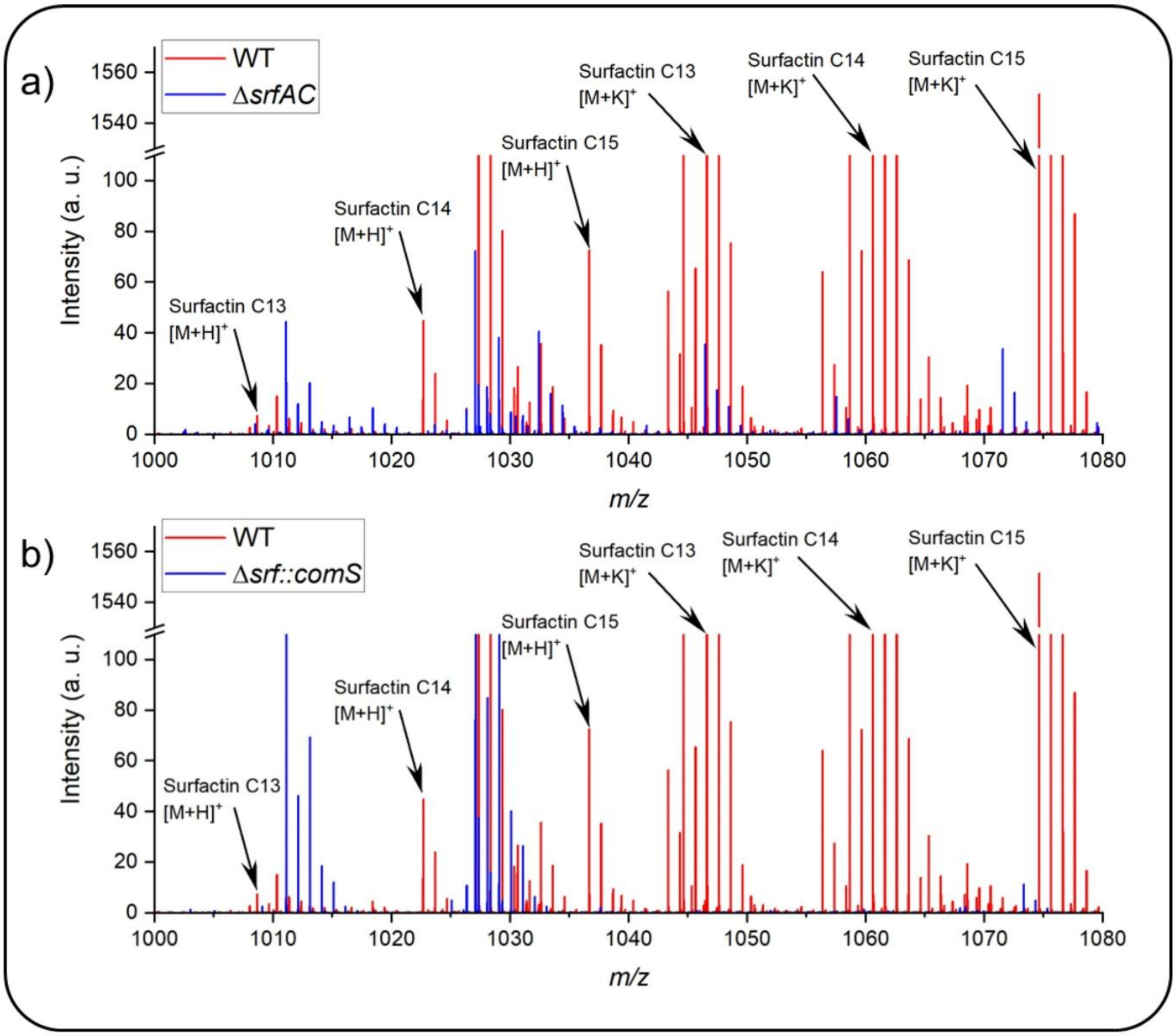

Figure S4: Summed MALDI-MS imaging spectra of *srf* operon deletion *srfAC* deletion strains, and wild type DK1042.

MALDI-MS spectra of wild type DK1042 (wt; red), and corresponding mutants (blue). Distinct ion adducts, including $[M+H]^+$, and $[M+K]^+$ species of the respective compounds are indicated with arrows. **(a-b)** Spectra from *srfAC* and *srf* operon deletion strains, respectively, reveal the complete loss of surfactin signals, confirming the absence of surfactin production in these backgrounds.

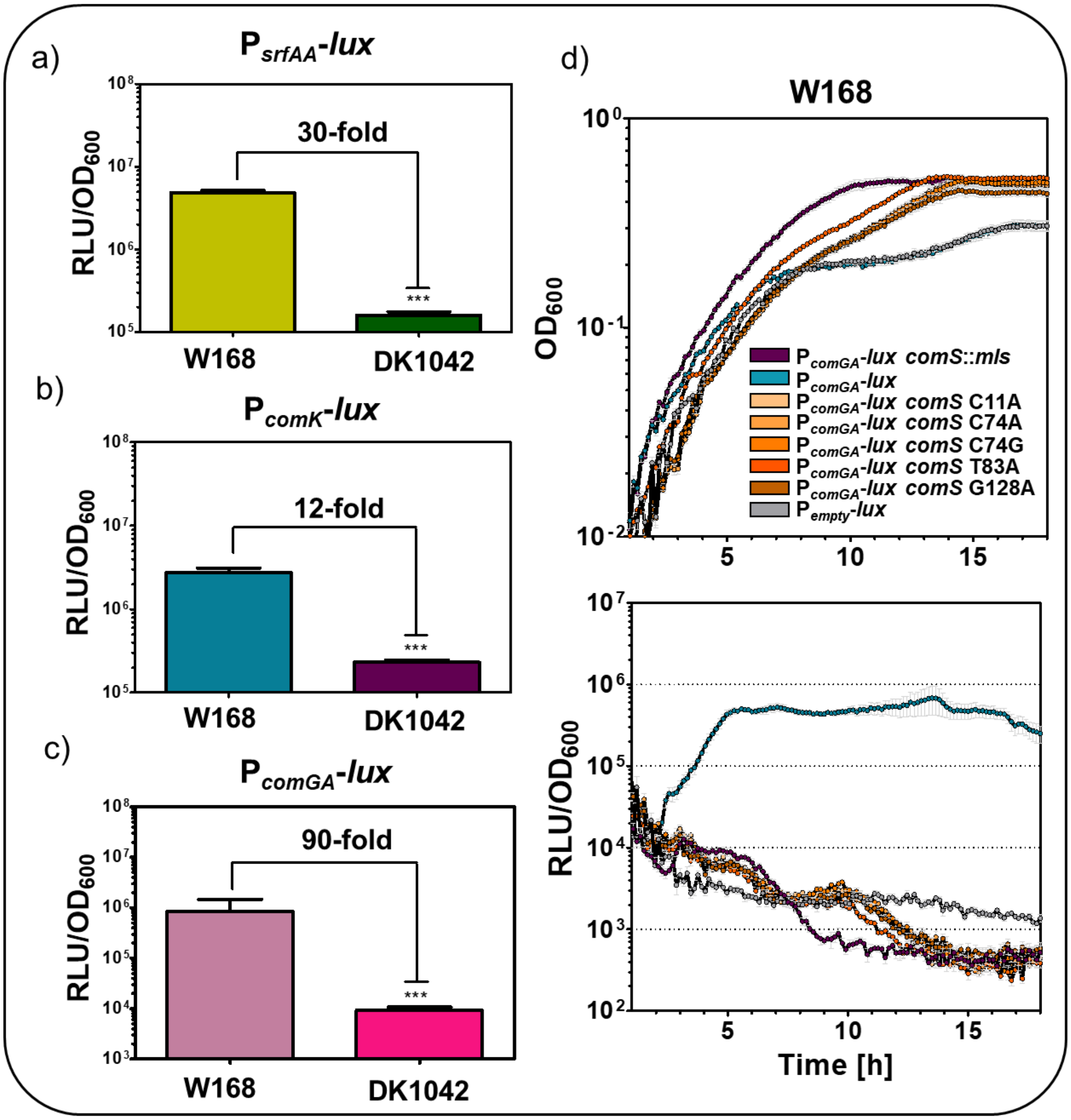

Figure S5: Comparative analysis of competence-associated promoters.

Comparison of **a)** P_{srfAA}-*lux*, **b)** P_{comK}-*lux*, and **c)** P_{comGA}-*lux* activity in the laboratory strain W168 and biofilm-forming DK1042 was shown as bar graphs of maximum RLU/OD_{600} values over time. Statistical significance as result of t-test performed as two sample assuming with unequal variances was indicated by: ns = not significant; * = p<0.05; ** = p<0.01; *** = p<0.001. **d)** Impact of *comS* point mutations on competence development in *B. subtilis* W168 the activity of P_{comGA}-lux in absence and presence of *comS* and the *comS* point mutations (C11A, C74A, C74G, T83A, and G128A) was monitored by measurement of relative luminescence units normalised to the corresponding OD_{600} values. P_{empty}-*lux* served as control, indicating background activity of the reporter strain. The standard deviation of the biological and technical triplicates was included as error bars to each time point of measurement.

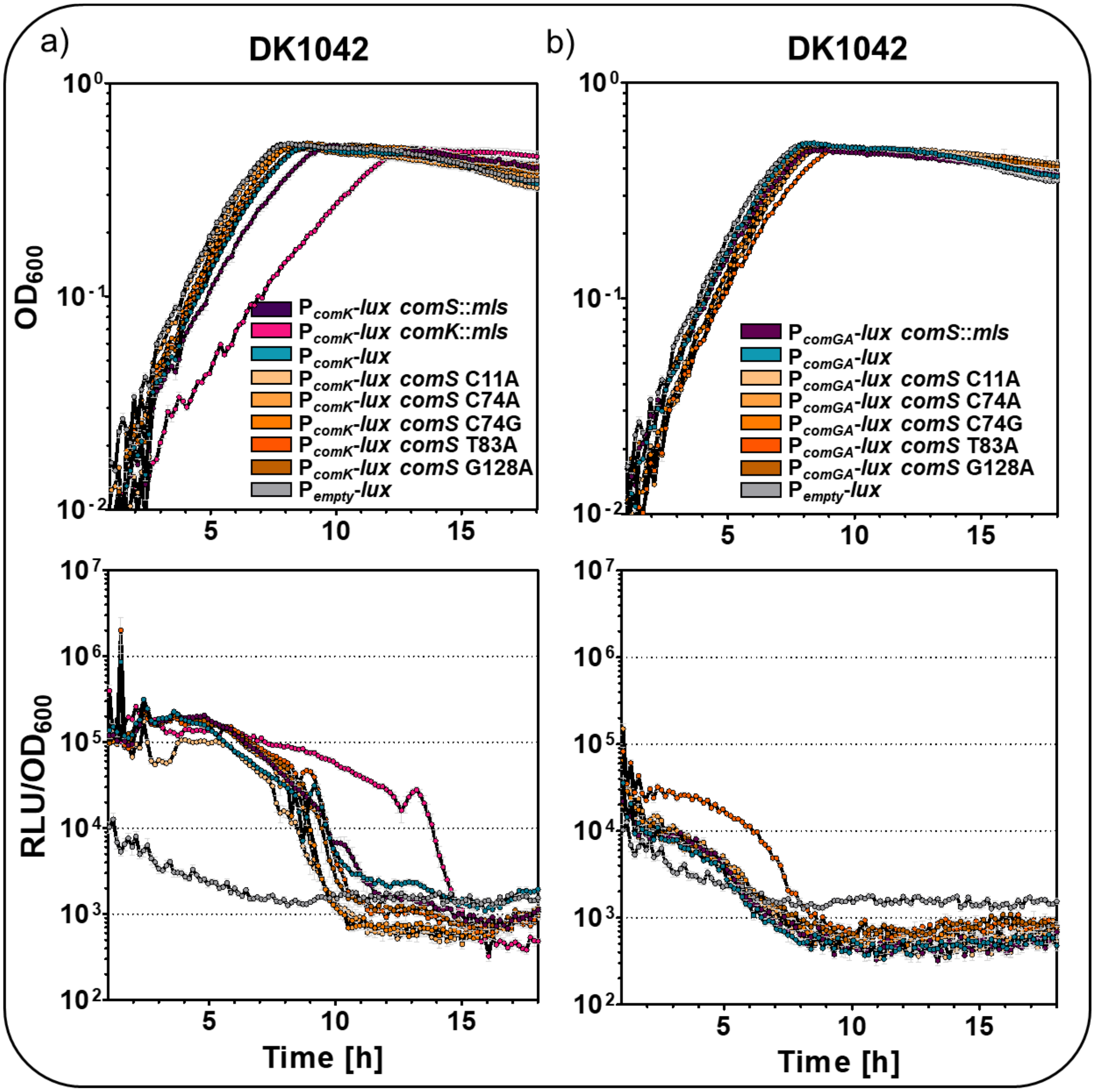

Figure S6: Impact of *comS* point mutations on competence development in *B. subtilis* DK1042.

The activity of **a)** P_{comK}-*lux* and **b)** P_{comGA}-*lux* in absence and presence of *comS* and *comK* deletion as well as the *comS* point mutations (C11A, C74A, C74G, T83A, and G128A) was monitored by measurement of relative luminescence units normalised to the corresponding OD_{600} values. P_{empty}-*lux* served as control, indicating background activity of the reporter strain. The growth was depicted as function of OD_{600} over time. The standard deviation of the biological and technical triplicates was included as error bars to each time point of measurement

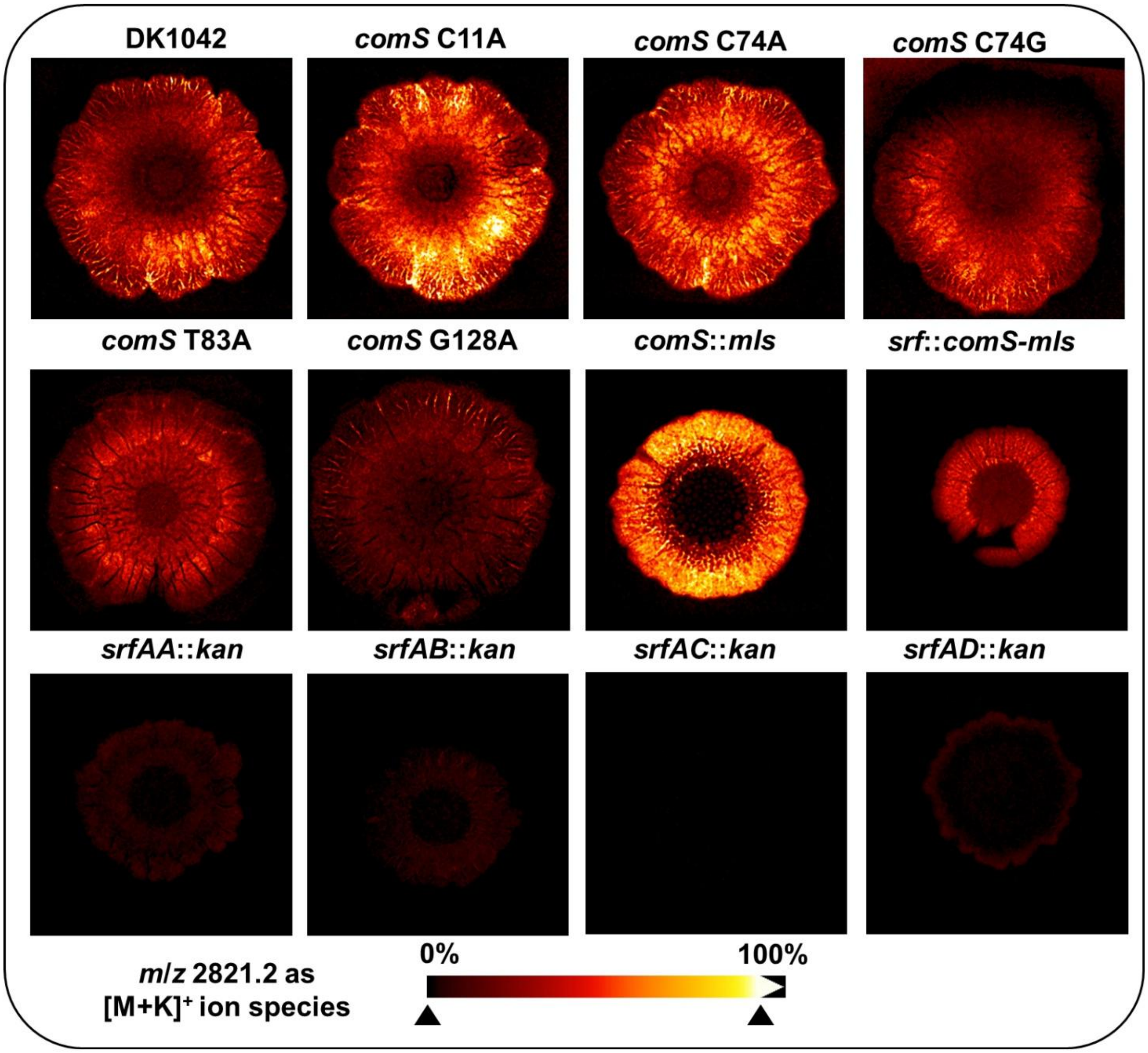

Figure S7: MALDI-MS images of SKF in *comS* and *srfAABCD* mutant biofilms.

Representative images of SKF production in wild type (DK1042), *comS* SNP strains (C11A, C74A, C74G, T83A, and G128A), and various *srf* mutants (*srfAA*::*kan*, *srfAB*::*kan*, *srfAC*::*kan*, *srfAD*::*kan*, *comS*::*mls*, *srf*::*comS-mls*) was displayed. The distribution of $[M+K]^+$ ion signals of SKF detected at *m*/*z* 2821.2 is presented. The scale bar indicates 1 cm.

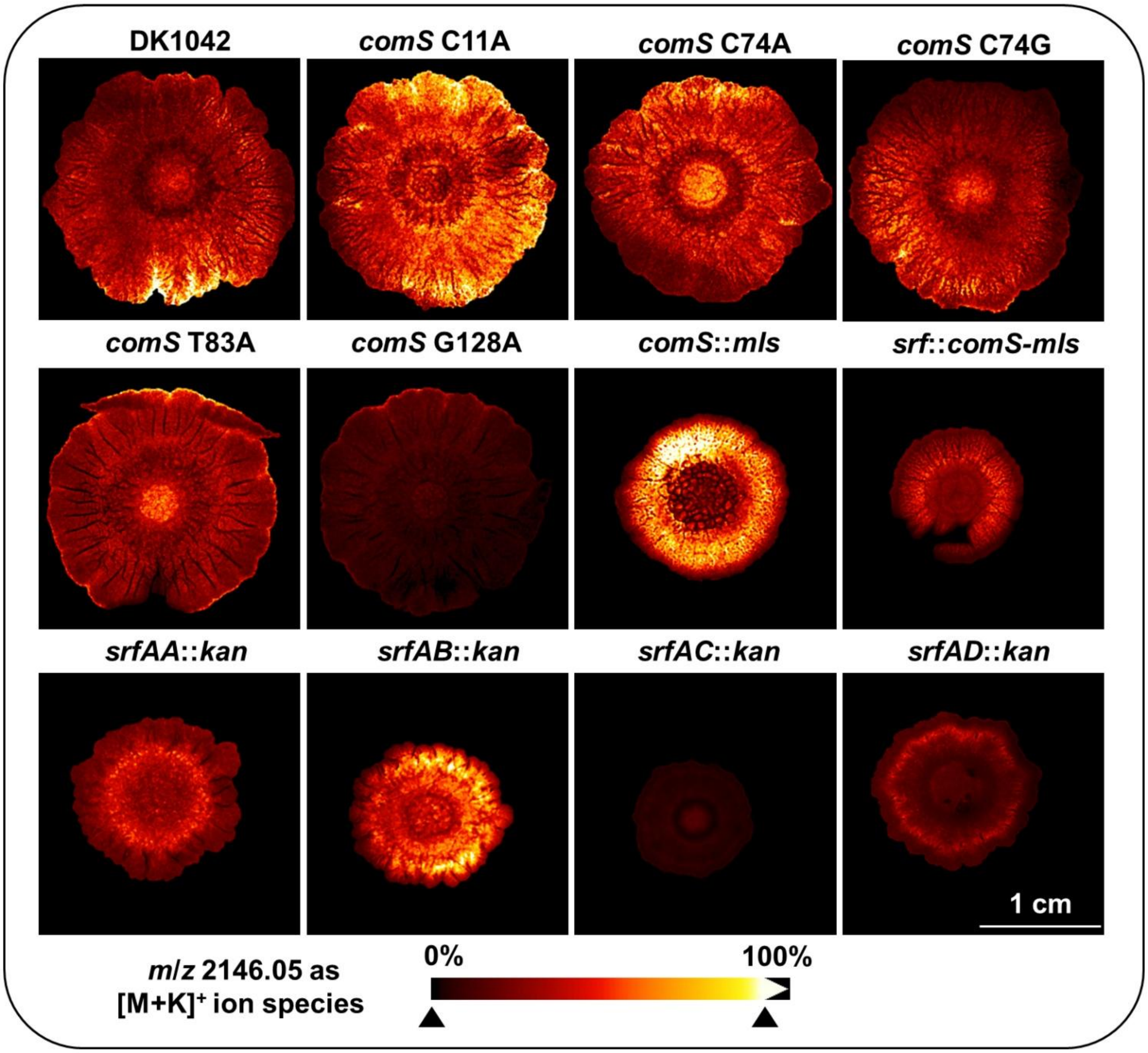

Figure S8: MALDI-MS images of EPE in *comS* and *srfAABCD* mutant biofilms.

Representative images of EPE production in wild type (DK1042), *comS* SNP strains (C11A, C74A, C74G, T83A, and G128A), and various *srf* mutants (*srfAA*::*kan*, *srfAB*::*kan*, *srfAC*::*kan*, *srfAD*::*kan*, *comS*::*mls*, *srf*::*comS-mls*) was displayed. The distribution of $[M+K]^+$ ion signals of EPE detected at *m*/*z* 2146.05 is presented. The scale bar indicates 1 cm.

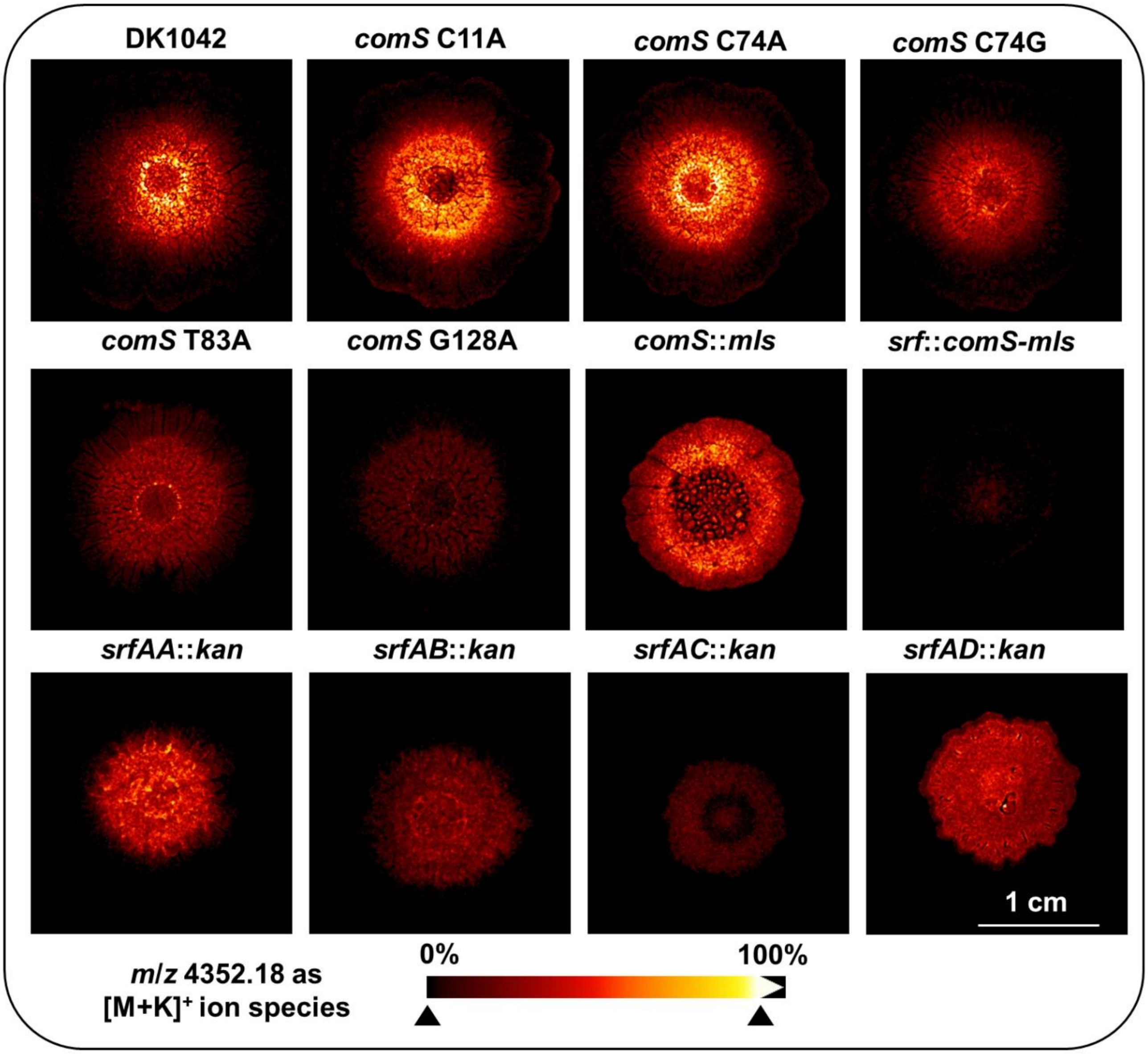

Figure S9: MALDI-MS images of SDP in *comS* and *srfAABCD* mutant biofilms.

Representative images of SDP production in wild type (DK1042), *comS* SNP strains (C11A, C74A, C74G, T83A, and G128A), and various *srf* mutants (*srfAA*::*kan*, *srfAB*::*kan*, *srfAC*::*kan*, *srfAD*::*kan*, *comS*::*mls*, *srf*::*comS-mls*) was displayed. The distribution of $[M+K]^+$ ion signals of SDP detected at *m*/*z* 4352.18 is presented. The scale bar indicates 1 cm.
